# Transformable Tumor Microenvironment‐Responsive Oxygen Vacancy‐Rich MnO_2_@Hydroxyapatite Nanospheres for Highly Efficient Cancer Sonodynamic Immunotherapy

**DOI:** 10.1002/advs.202414162

**Published:** 2025-02-17

**Authors:** Minxing Li, Qiyu Liu, Songzuo Xie, Desheng Weng, Jinjun He, Xinyi Yang, Yuanyuan Liu, Jinqi You, Jinghao Liao, Peng Wang, Xihong Lu, Jingjing Zhao

**Affiliations:** ^1^ State Key Laboratory of Oncology in South China Guangdong Provincial Clinical Research Center for Cancer Collaborative Innovation Center for Cancer Medicine, Department of Biotherapy Sun Yat‐Sen University Cancer Center Guangzhou 510060 P. R. China; ^2^ The Key Lab of Low‐carbon Chem & Energy Conservation of Guangdong Province School of Chemistry Sun Yat‐Sen University Guangzhou 510275 P. R. China; ^3^ Department of Emergency Medicine Sun Yat‐sen Memorial Hospital Sun Yat‐sen University Guangzhou 510120 P. R. China

**Keywords:** immunotherapy, reactive oxygen species, sonodynamic therapy, sonosensitizers, tumor microenvironment

## Abstract

Despite the promise of sonodynamic therapy (SDT)‐mediated immunotherapy, the anticancer efficacy of current sonosensitizers is greatly limited by the immunosuppressive tumor microenvironment (TME) and their inability to selectively respond to it. Herein, oxygen vacancy‐rich MnO_2_@hydroxyapatite (Ca_10_(PO_4_)_6_(OH)_2_) core–shell nanospheres (denoted as O_v_‐MO@CPO) as an advanced TME‐responsive sonosensitizer for sonodynamic immunotherapy is demonstrated. The O_v_‐MO@CPO maintains its structural integrity under neutral conditions but dissolves the pH‐sensitive hydroxyapatite shell under acidic TME to release active oxygen vacancy‐rich MnO_2_ core, which reinvigorates H_2_O_2_ consumption and hypoxia alleviation due to its catalase‐like activity. Furthermore, the introduced oxygen vacancies optimize the electronic structure of O_v_‐MO@CPO, with active electronic states near the Fermi level and higher d‐band center. It results in accelerated electron‐hole pair separation and lower catalytic energy barriers to boost ultrasound (US)‐initiated ROS production. These multimodal synergistic effects effectively reverse the immunosuppressive tumor microenvironment, inhibiting tumor growth and metastasis in 4T1 tumor‐bearing mice. No evident toxic effects are observed in normal mouse tissues. Additionally, when combined with an immune checkpoint inhibitor, O_v_‐MO@CPO‐mediated SDT further improves the effectiveness of immunotherapy. This work affords a new avenue for developing TME‐dependent sonosensitizers for SDT‐mediated immunotherapy.

## Graphical abstract

Transformable tumor microenvironment‐responsive oxygen vacancy‐rich MnO_2_@hydroxyapatite core–shell nanospheres are designed as a robust sonosensitizer for sonodynamic immunotherapy, which can effectively reverse the immunosuppressive tumor microenvironment, resulting in a satisfactory antitumor efficacy.

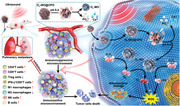

## 1 Introduction

Cancer is one of the leading causes of mortality in every country worldwide, posing a severe threat to human health.^[^ ^1^, ^2^ ^]^ Immunotherapy is a current hotspot in oncological research, which has revolutionized cancer treatment by leveraging the body's immune system to fight tumors.^[^ ^3^, ^4^, ^5^ ^]^ However, the clinical efficacy of immunotherapy is limited, with the immunosuppressive tumor microenvironment (TME) being a critical barrier.^[^ ^6^, ^7^ ^]^ The TME is characterized by abundant immunosuppressive cells, including M2 macrophages, myeloid‐derived suppressor cells (MDSCs), and regulatory T cell (Treg) cells, which significantly inhibit the activation of immune cells.^[^ ^8^, ^9^, ^10^, ^11^ ^]^ Furthermore, the unique properties of TME, such as acidic pH levels, hypoxia conditions, and high levels of endogenous H_2_O_2_ further downplay antitumor immune responses.^[^ ^12^, ^13^, ^14^, ^15^, ^16^ ^]^ Consequently, arousing immune cells and modulating TME are key to improving the antitumor effects of immunotherapy. Currently, many studies are investigating effective strategies to convert M2 macrophages into tumoricidal M1 cells. For example, the nanoparticle/bacteria complex (Ec‐PR848) system can be used for targeted drug delivery to tumors and macrophage polarization. When combined with PLGA‐DOX, which induces immunogenic cell death (ICD), this system enhanced the polarization of M2 macrophages to the M1 phenotype, activated antitumor immune responses, and inhibited tumor growth.^[^ ^17^ ^]^ However, most approaches to macrophage repolarization are still in the basic research phase and have not yet been effectively applied in clinical practice. Therefore, reprogramming M2 macrophages into M1 cells remains a significant challenge for cancer immunotherapy. Extensive investigations have suggested that in addition to providing oxidative stress that causes toxicity, reactive oxygen species (ROS) are essential signaling molecules in activating systemic antitumor immunity: i) ROS plays an important role in inducing the maturation of dendritic cells (DCs) and reprogramming M2 macrophages to M1 type; ii) ROS can induce ICD of cancer cells to release damage‐associated molecular patterns (DAMPs), like calreticulin (CRT), heat shock proteins (HSPs), adenosine triphosphate (ATP), heat shock protein 70 (HSP70) and high mobility group box 1 (HMGB1).^[^ ^18^, ^19^, ^20^, ^21^, ^22^ ^]^ These results collectively energize systemic immune response.

In recent years, many emerging ROS‐based therapies, such as photodynamic therapy (PDT)^[^ ^23^, ^24^ ^]^ and chemodynamic therapy (CDT),^[^ ^25^, ^26^ ^]^ can facilely regulate ROS generation. Among them, sonodynamic therapy (SDT) utilizes sonosensitizers to produce localized ROS under low‐intensity ultrasound (US) irradiation, which possesses the advantages of elevated subcutaneous penetration (up to 10 cm), noninvasiveness, high spatial precision, and negligible side effects.^[^ ^27^, ^28^ ^]^ More importantly, SDT has shown promising results in eliciting an immune response.^[^ ^29^, ^30^, ^31^ ^]^ For instance, a liposome‐modified hematoporphyrin monomethyl ether/imiquimod (HMME/R837@Lip) sonosensitizer was designed to boost the release of tumor‐associated antigens derived from SDT, promoting DCs maturation (CD80^+^CD86^+^ DCs) and cytokine secretion, thus killing tumor cells.^[^ ^32^ ^]^ Additionally, a manganese‐protoporphyrin sonosensitizer modified with folate‐liposomes (FA‐MnPs) induced an SDT‐mediated immune response, re‐polarizing M2 macrophages to the antitumor M1 phenotype and simultaneously activating DCs, T lymphocytes, and natural killer cells, consequently inhibiting tumor growth.^[^ ^33^ ^]^ Moreover, some new sonosensitizers with special physicochemical properties can achieve additional oxygen supply, H_2_O_2_ depletion, and ions overload to effectively modulate TME, thus further enhancing tumor immunotherapy.^[^ ^34^, ^35^, ^36^ ^]^ For example, PEGylated CoFe_2_O_4_ nanoflowers with multivalent elements (Co^2+/3+^, Fe^2+/3+^) possessed catalase‐like activity to react with endogenous H_2_O_2_ to achieve high O_2_ level, which enhanced the production of ^1^O_2_ for SDT and alleviated immunosuppression of TME.^[^ ^37^ ^]^ Although these inspiring achievements, these sonosensitizers usually possess “always on” pharmacological activities, rather than specific release in the disease microenvironment, potentially reducing therapeutic effectiveness and causing damage to healthy tissues.^[^ ^38^, ^39^, ^40^ ^]^ Therefore, it is urgently necessary to explore novel sonosensitizers to match SDT‐mediated immunotherapy, which not only have excellent ROS generation and additional TME modulation capability but also can maintain their integrity under non‐targeted physiological conditions and respond to specific microenvironments.

**Scheme 1:**
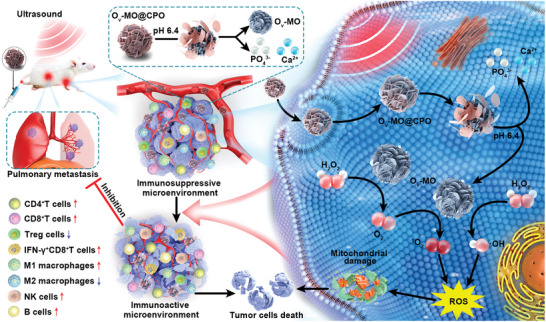
Schematic mechanism of O_v_‐MO@CPO for sonodynamic tumor therapy.

In this study, we engineered transformable TME‐responsive oxygen vacancy‐rich MnO_2_@hydroxyapatite (Ca_10_(PO_4_)_6_(OH)_2_) core–shell nanospheres (denoted as O_v_‐MO@CPO) as a robust sonosensitizer for highly efficient SDT‐mediated immunotherapy (Scheme 1). Hydroxyapatite (CPO), with its acidic degradation ability, acts as a pH‐responsive coating that remains inert under a neutral normal microenvironment to protect O_v_‐MO core. Under acidic TME, it releases an active O_v_‐MO core for US‐initiated ROS generation and conversion of H_2_O_2_ to O_2_ due to the catalase‐like property of multivalent Mn ions. Moreover, benefiting from the existence of oxygen vacancies, the electronic structure of released O_v_‐MO is optimized with many active electronic states around the Fermi level and higher d‐band center, which considerably reduce the energy barriers of catalytic reaction, thereby further boosting the ROS generation. As expected, the combination of ROS generation and hypoxia relief in the TME enabled O_v_‐MO@CPO to effectively reprogram tumor‐associated macrophages (TAMs) from M2 to M1 phenotype, increasing the infiltration of CD4^+^T cells, CD8^+^T cells, NK cells, and B cells in the tumor, while significantly reducing the infiltration of Treg cells, thereby greatly reversing the immunosuppressive TME. Furthermore, in combination with an immune checkpoint blockade therapy, the O_v_‐MO@CPO‐mediated immunotherapy efficacy is further enhanced by great infiltration of CD4^+^T cells and CD8^+^T cells. Consequently, the O_v_‐MO@CPO nanospheres achieve ultrahigh antitumor efficacy both in vitro and in vivo with negligible cytotoxicity at therapeutic doses.

## 2 Results and Discussion

**Figure 1:**
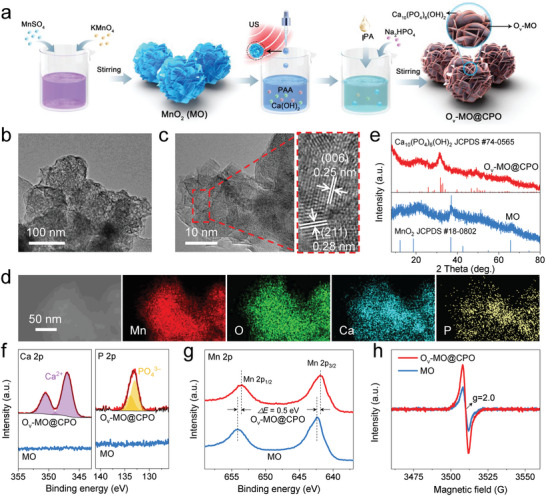
a) Diagram depicting the synthesis procedure of MO and O_v_‐MO@CPO nanospheres. b) TEM image, c) HRTEM image and d) EDS mapping of O_v_‐MO@CPO. e) XRD patterns, f) Ca 2p core‐level XPS spectra, P 2p core‐level XPS spectra, g) Mn 2p core‐level XPS spectra and h) ESR spectra of MO and O_v_‐MO@CPO.

O_v_‐MO@CPO nanospheres were obtained via a two‐step synthetic process. First, MnO_2_ (MO) nanospheres with a size of 100–150 nm were prepared using a liquid precipitation method (Figure S1a, Supporting Information). Then, the polyacrylic acid (PAA) with abundant carboxyl groups and Ca(OH)_2_ were introduced into the solution of these MO nanospheres to reacted to PAA‐Ca^2+^. Upon the addition of isopropanol (IPA), the PAA‐Ca^2+^ self‐assembled around the surface of MO via the electrostatic interaction. Subsequently, Na_2_HPO_4_ was added as a phosphate anion source to produce a hydroxyapatite coating to form O_v_‐MO@CPO nanospheres (**Figure** **1**a). Scanning electron microscopy (SEM) image in Figure S1b (Supporting Information) shows that the O_v_‐MO@CPO sample has a similar nanosphere‐like structure. In addition, according to transmission electron microscopy (TEM) images (Figure S2a, Supporting Information), MO nanospheres are made up of many intersecting flakes with a thickness of only 1–3 nm, demonstrating its large specific surface area. Meanwhile, the morphology of O_v_‐MO@CPO has no obvious change compared with that of MO, indicating that the CPO shell is ultrathin and uniformly covered on the surface of nanosheets (Figure 1b; Figure S2b, Supporting Information). In addition, the MO possesses a layer spacing of 0.70 nm and a lattice spacing of 0.25 nm in a high‐resolution TEM (HRTEM) image, matched well with the (002) and (006) planes of layered birnessite δ‐MnO_2_ (JCPDS No. 18–0802), respectively (Figure S3, Supporting Information).^[^ ^41^ ^]^ As for O_v_‐MO@CPO, it shows the presence of (006) planes of δ‐MnO_2_ and (211) planes (0.28 nm) of hydroxyapatite (Ca_10_(PO_4_)_6_(OH)_2_, JCPDS No. 74–0565) at the edge of flakes (Figure 1c; Figure S4, Supporting Information), verifying the successful synthesis of O_v_‐MO@CPO core–shell structure.^[^ ^42^ ^]^ The corresponding diffraction spots in the selected area electron diffraction (SAED) pattern of two materials further support this viewpoint (Figure S5, Supporting Information). Additionally, the uniform distribution of Mn, O, Ca, and P elements along the O_v_‐MO@CPO architecture in energy‐dispersive X‐ray spectroscopy (EDS) mapping illustrates the precise modification of the hydroxyapatite coating (Figure 1d). The crystalline structures of MO and O_v_‐MO@CPO were identified by X‐ray diffraction (XRD). The XRD pattern of MO in Figure 1e agrees with δ‐MnO_2_ (JCPDS No. 18–0802), displaying its nanoscale crystallinity.^[^ ^43^, ^44^ ^]^ Interestingly, the XRD pattern of O_v_‐MO@CPO appears new diffraction peaks corresponding to Ca_10_(PO_4_)_6_(OH)_2_ (JCPDS No. 74–0565), while the peak intensities of δ‐MnO_2_ are obviously weakened. This suggests that the modification of CPO coating results in the partial amorphization of MnO_2_ core. X‐ray photoelectron spectroscopy (XPS) measurements were conducted to further examine the influence of CPO coating. Compared with MO, both the Ca 2p and P 2p spectra of O_v_‐MO@CPO exhibit a doublet peak, which corresponds to Ca^2+^ and PO_4_^3–^ in hydroxyapatite (Figure 1f).^[^ ^45^ ^]^ In Mn 3s spectra, O_v_‐MO@CPO possesses a higher binding energy separation of 4.9 eV than MO (4.5 eV), indicating that the dominated valence state of Mn species in MO is +4 while Mn^3+^ and Mn^4+^ coexist in O_v_‐MO@CPO (Figure S6, Supporting Information).^[^ ^46^ ^]^ Obviously, the peak positions of Mn 2p_3/2_ and Mn 2p_1/2_ in O_v_‐MO@CPO exhibit a negative shift of 0.5 eV, which also signifies its increased low‐valent Mn species (Figure 1g). It may be due to the formation of oxygen vacancies during the synthesis of CPO coating, thus resulting in a partial reduction of Mn species, which is confirmed by electron spin resonance (ESR). Consistent with the previous results, the O_v_‐MO@CPO shows a stronger peak intensity at *g* = 2.0 (typical signal of oxygen vacancies) than MO, clearly implying that oxygen vacancies are presented in O_v_‐MO@CPO (Figure 1h).^[^ ^47^, ^48^ ^]^ All these observations verify that O_v_‐MO@CPO nanospheres are composed by CPO shell and oxygen vacancy‐modified MnO_2_ (O_v_‐MO) core.

**Figure 2:**
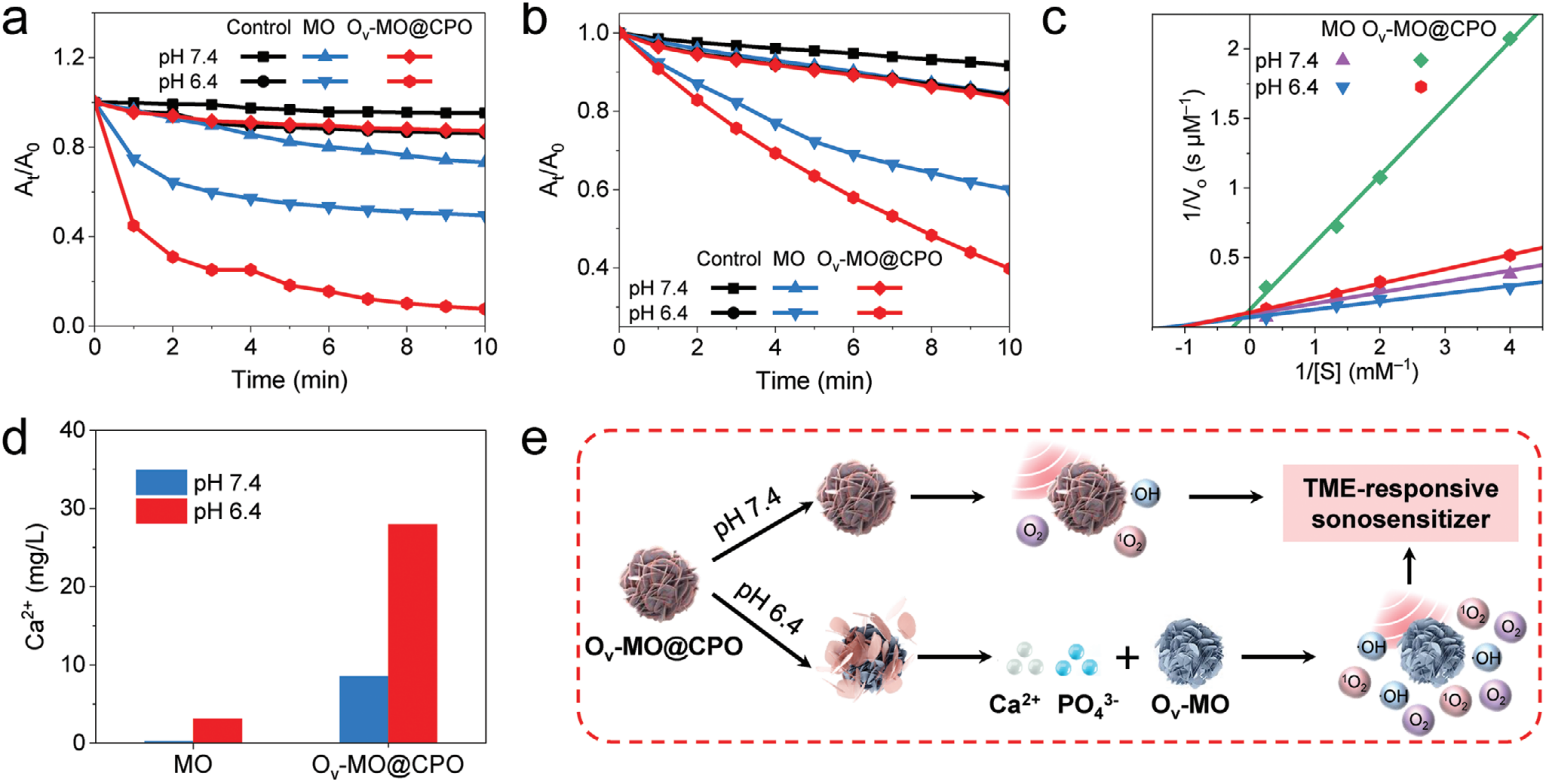
The pH‐dependent behavior of O_v_‐MO@CPO nanospheres (0.1 mg mL^−1^) in PBS solution. a) ·OH generation detected by MB and b) ^1^O_2_ generation detected by DPBF of control, MO, and O_v_‐MO@CPO by US irradiation at different pH values. c) The detection of O_2_ production of MO and O_v_‐MO@CPO with H_2_O_2_ addition (0.5 mm). d) Ca^2+^ release of MO and O_v_‐MO@CPO after standing at pH 6.4 and pH 7.4 for 24 h, respectively. e) Illustration showing mechanisms of O_v_‐MO@CPO nanospheres.

To assess the sonodynamic performance, MO and O_v_‐MO@CPO were incubated in the neutral (pH 7.4, simulating a normal microenvironment) and acidic (pH 6.4, simulating TME) PBS solution, respectively. The ROS yield under US irradiation was then evaluated using UV–vis absorbance measurements and probes. First, methylene blue (MB) was employed to detect the generation of hydroxyl radicals (·OH), which can react to produce MB‐OH with low absorbance (Figure S7, Supporting Information).^[^ ^49^ ^]^ Upon US irradiation for 10 min at pH 7.4, MO and O_v_‐MO@CPO consumed only 26.8% and 12.7% of MB, respectively. In contrast, at pH 6.4, O_v_‐MO@CPO exhibited a 7.3‐fold higher decrease rate of MB compared to MO (1.9‐fold) (**Figure** **2**a). Meanwhile, a similar trend was found when 1,3‐diphenylisobenzofuran (DPBF) was employed as a molecular probe to monitor the formation of singlet oxygen (^1^O_2_) (Figure S8, Supporting Information).^[^ ^50^ ^]^ As shown in Figure 2b, MO and O_v_‐MO@CPO achieved a comparable decline of the DPBF peak intensity at pH 7.4. However, a sharp contrast for a decrease rate of DPBF was observed at pH 6.4 between two materials. These data confirm that O_v_‐MO@CPO possesses superior pH‐responsive sonodynamic properties. In addition to its special ROS yield ability, O_v_‐MO@CPO was also demonstrated to exhibit pH‐responsive catalase‐like activity, which can decompose intracellular H_2_O_2_ into O_2_. The dissolved O_2_ increments of samples at different concentrations of H_2_O_2_ (0.25, 0.50, 0.75, and 4 mm) and pH values (7.4 and 6.4) are measured in real‐time, respectively (Figure S9, Supporting Information). According to Michaelis–Menten equation and Lineweaver‐Burk plot (double‐reciprocal, Figure 2c), the catalase‐like properties of MO and O_v_‐MO@CPO are quantitively compared (Table S1). To be specific, the Michaelis constant (*K_m_*) of O_v_‐MO@CPO at pH 6.4 is 0.98 mm, which is a little different from that of MO at pH 7.4 (0.87 mm) and pH 6.4 (0.80 mm), reflecting that the binding affinity for the H_2_O_2_ of two samples at pH 7.4 is similar, and the binding affinity of MO is less affected by pH. On the contrary, the *K_m_* of O_v_‐MO@CPO at pH 6.4 increases significantly to 3.8 mm, confirming its pH‐responsive catalase‐like activity. The ratio of catalytic constant (*k_cat_*) to *K_m_* (catalytic efficiency, *k_cat_*/*K_m_*) also well supports this viewpoint. The *k_cat_*/*K_m_* of O_v_‐MO@CPO at pH 6.4 is 4.7 times that at pH 7.4, while the *k_cat_*/*K_m_* of MO at pH 6.4 is only 1.4 times that at pH 7.4. The slightly weaker *V_max_* and *k_cat_* of O_v_‐MO@CPO than MO is attributed to its lower amount of Mn species at the same test concentration due to the introduction of hydroxyapatite coverage. Subsequently, to identify the pH‐responsive mechanism of O_v_‐MO@CPO, MO, and O_v_‐MO@CPO nanospheres were immersed in PBS solutions with different pH values for 24 h for inductively coupled plasma atomic emission spectrometry (ICP‐AES), respectively. As displayed in Figure 2d, a significantly higher Ca^2+^ concentration of 28.0 mg L^−1^ was detected in the supernatant of O_v_‐MO@CPO solution at pH 6.4 than that at pH 7.4 (8.6 mg L^−1^), indicating that CPO shell remains stable at pH 7.4, but will dissolve at pH 6.4. Namely, the proposed pH‐responsive mechanism of O_v_‐MO@CPO is presented in Figure 2e. Briefly, under neutral conditions, the inert CPO shell isolates the MnO_2_ core, which is unable to fully contact the reactants to produce ROS and O_2_. Under acidic conditions, CPO shell disintegrates and O_v_‐MO core is exposed to recover its catalytic performance, thus achieving TME‐responsive SDT.

**Figure 3:**
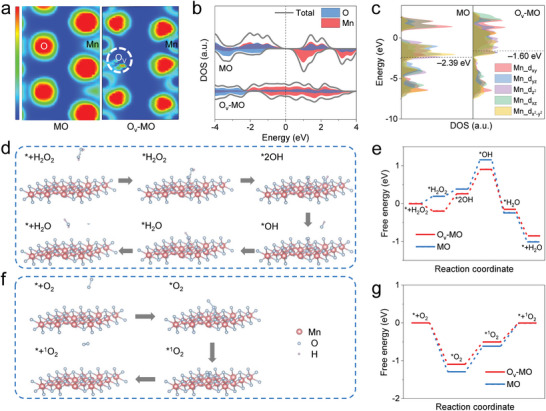
a) ELF, b) DOS, and c) computed Mn 3d PDOS of MO and O_v_‐MO. d) ·OH generation catalytic path of MO and O_v_‐MO and e) corresponding Gibbs free energy diagram. f) ^1^O_2_ generation catalytic path of MO and O_v_‐MO and g) corresponding Gibbs free energy diagram.

Notably, the O_v_‐MO core released by O_v_‐MO@CPO exhibits more efficient ROS generation ability than pure MO, which may be attributed to the role of oxygen vacancies. Density functional theory (DFT) calculations were conducted to elucidate the influential mechanism of oxygen vacancies on catalytic activities of O_v_‐MO core. The optimized model structures for MO and O_v_‐MO were established (Figure S10, Supporting Information). First, the electron localization function (ELF) of two models was simulated and displayed in **Figure** **3**a. Upon the introduction of oxygen vacancies, the electrons of Mn atoms are transferred to adjacent O atoms, resulting in a continuous electronic density and increased electron delocalization.^[^ ^51^, ^52^ ^]^ The abundant delocalized electrons form channels connecting states below and across the Fermi level, which can be further confirmed by density of states (DOS) analysis (Figure 3b). MO has an obvious bandgap, whereas O_v_‐MO features numerous active electronic states around the Fermi level. This special electronic structure of O_v_‐MO facilitates the excitation of more electron‐hole pairs under US irradiation and accelerates their separation due to ameliorative electron transfer ability. Additionally, the d‐band center of O_v_‐MO shifts positively (from −2.39 to −1.60 eV), which favors the adsorption of intermediate O to boost catalytic reaction (Figure 3c).^[^ ^53^, ^54^ ^]^ As proof, the catalytic pathways and corresponding Gibbs free energy of ROS generation were simulated.^[^ ^55^, ^56^ ^]^ As depicted in Figure 3d,e, H_2_O_2_ is first adsorbed on the Mn site of MO and O_v_‐MO with adsorption energies of 0.20 and −0.19 eV, respectively. The negative Gibbs free energy in O_v_‐MO implies that this step is thermodynamically feasible, which makes H_2_O_2_ easily adsorbed on the surface. The absorbed substrate (*H_2_O_2_) then cleaves into two *OH groups. After that, a free hydroxyl radical is desorbed from the Mn site, which is the rate‐determining step. The energy barrier of O_v_‐MO is 0.64 eV, lower than that of MO (0.77 eV). Finally, the residual *OH can absorb H^+^ due to acidic TME to form *H_2_O, followed by desorption. Based on this, benefiting from enhanced adsorption and reduced energy barrier, O_v_‐MO can rapidly produce ·OH. Similarly, the enhancement mechanism of ^1^O_2_ yield was also studied (Figure 3f,g).^[^ ^57^, ^58^, ^59^ ^]^ The surface‐adsorbed O_2_ on MO undergoes two uphill energy barriers of 0.67 eV from *O_2_ to *^1^O_2_ (rate‐determining step) and 0.62 eV from *^1^O_2_ to ^1^O_2_. As for O_v_‐MO, the presence of oxygen vacancies enables it lower energy barriers of 0.59 and 0.50 eV, respectively, enhancing the ^1^O_2_ production. In conclusion, the oxygen vacancies modulate the electronic structure of O_v_‐MO core, which activates the catalytic performance of O_v_‐MO@CPO to efficiently produce ROS.

**Figure 4:**
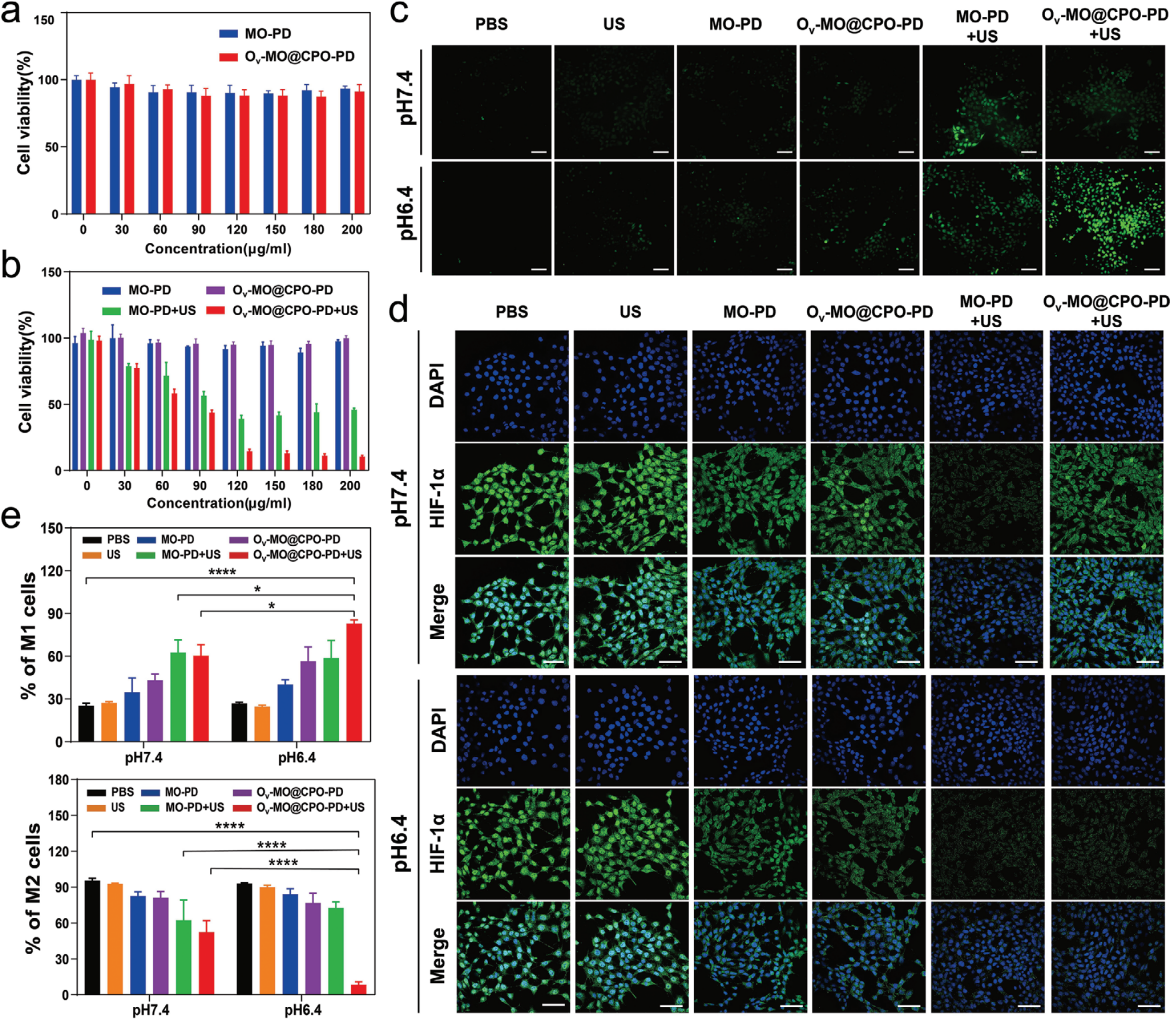
In vitro sonodynamic therapeutic efficacy of O_v_‐MO@CPO‐PD nanospheres. a) Cell viability of HUVECs incubated with MO‐PD and O_v_‐MO@CPO‐PD at different concentrations. b) Cell viability of 4T1 cells incubated with MO‐PD and O_v_‐MO@CPO‐PD at different concentrations with or without US (1 MHz, 2 W cm^−^^2^, 2 min). c) Confocal fluorescence images of 4T1 cells stained with DCFH‐DA to detect intracellular ROS levels. Scale bar: 500 µm. d) immunofluorescence analysis of HIF‐1α expression of 4T1 cells. Scale bar: 50 µm. e) Flow cytometry quantification of CD80 (M1 macrophage marker) and CD206 (M2 macrophage marker) expression on Raw264.7 cell. ^*^ *p* < 0.05, ^****^ *p* < 0.0001.

Given the synergistic effects of O_v_‐MO@CPO under US irradiation, we explored its potential therapeutic effects in vitro. First, to enhance their biocompatibility and dispersion stability, the surface of MO and O_v_‐MO@CPO were modified with 1,2‐distearoyl‐sn‐glycero‐3‐phosphoethanolamine‐N‐[methoxy(polyethylene glycol)‐2000] (mPEG‐DSPE) (denoted as MO‐PD and O_v_‐MO@CPO‐PD, respectively). The cytotoxicity of O_v_‐MO@CPO‐PD was initially detected by CCK‐8 assay. The cell viability of human umbilical vein endothelial cells (HUVECs) and LO2 cells incubated with O_v_‐MO@CPO‐PD did not significantly change, even at high concentrations up to 200 µg mL^−1^ (**Figure** **4**a; Figure S12a, Supporting Information), indicating that these O_v_‐MO@CPO‐PD nanospheres exhibit minimal cytotoxicity toward normal cells. However, both O_v_‐MO@CPO‐PD and MO‐PD exhibited cytotoxic effects on 4T1 and Hepa1‐6 tumor cells when exposed to ultrasound, with the O_v_‐MO@CPO‐PD+US group showing more pronounced cytotoxic effects than the MO‐PD+US group (Figure 4b; Figure S12b, Supporting Information). These results demonstrate that O_v_‐MO@CPO‐PD plays a significant role in killing tumor cells under US irradiation and possesses good biocompatibility. An intracellular ROS assay was conducted on 4T1 cells to investigate the potential mechanism underlying the decreased cell viability of O_v_‐MO@CPO‐PD. ROS generation was evaluated under both physiologically neutral and pathologically acidic conditions using DCFH‐DA by confocal fluorescence microscopy.^[^ ^60^, ^61^ ^]^ The green fluorescence, indicative of intracellular ROS levels, was notably absent in the O_v_‐MO@CPO‐PD and US groups at both pH of 6.4 and 7.4. However, cells treated with both O_v_‐MO@CPO‐PD and US irradiation displayed green fluorescence, with the O_v_‐MO@CPO‐PD+US group at pH 6.4 showing significantly stronger green fluorescence than at pH 7.4 (Figure 4c). These observations indicate that O_v_‐MO@CPO‐PD, as a TME‐responsive sonosensitizer, is capable of producing ROS under US irradiation in an acidic environment. Additionally, to demonstrate the positive effect of O_v_‐MO@CPO‐PD on alleviating hypoxia, the intracellular oxygen content was assessed using immunofluorescence with HIF‐1α, an indicator of intracellular O_2_ level.^[^ ^62^, ^63^ ^]^ As displayed in Figure 4d, no significant difference in HIF‐1α expression was observed between the O_v_‐MO@CPO‐PD and O_v_‐MO@CPO‐PD+US groups at pH 7.4. However, at pH 6.4, HIF‐1α expression was dramatically lower in the O_v_‐MO@CPO‐PD+US group compared to the O_v_‐MO@CPO‐PD group, revealing that US‐irradiated O_v_‐MO@CPO‐PD can greatly improve the anoxic microenvironment of tumors by releasing O_2_ in an acidic environment.

Next, the capacity of O_v_‐MO@CPO‐PD to repolarize M2 macrophages to the M1 phenotype was evaluated in vitro. The murine macrophage RAW264.7 cells were stimulated to the M2‐like phenotype by incubating them with murine interleukin‐4 (IL‐4) for 24 h.^[^ ^64^, ^65^ ^]^ M2‐like RAW264.7 cells were then cultured with various formulations, followed by analysis using flow cytometry. Expressions of CD80 (a marker of M1) and CD206 (a marker of M2) were used to identify macrophage phenotypes.^[^ ^66^, ^67^ ^]^ According to the flow cytometry results, the number of M1 macrophages (CD11b^+^CD80^+^) increased, while the number of M2 macrophages (CD11b^+^CD206^+^) decreased in the O_v_‐MO@CPO‐PD+US group compared to the O_v_‐MO@CPO‐PD group. In addition, after O_v_‐MO@CPO‐PD and ultrasound irradiation treatment, the number of M1 macrophages obviously increased, while the number of M2 macrophages visibly decreased at pH 6.4 compared to pH 7.4 (Figure 4e; Figure S13, Supporting Information). These results confirm that O_v_‐MO@CPO‐PD can efficiently repolarize M2 macrophages to the M1 phenotype in an acidic environment under US irradiation, leading to a desirable antitumor immune efficacy.

**Figure 5:**
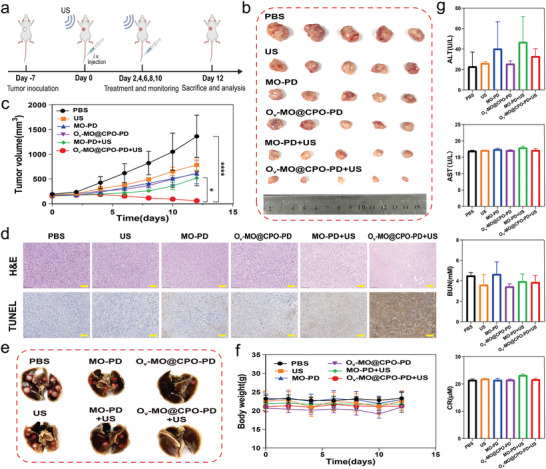
In vivo antitumor efficacy of O_v_‐MO@CPO‐PD in 4T1 tumor‐bearing mice. a) Schematic illustration of the treatment procedure for O_v_‐MO@CPO‐PD mediated antitumor efficacy in vivo. b) Representative photographs of tumors at the end of antitumor treatments. c) Tumor growth curves of mice after different treatments. d) H&E and TUNEL analyses of tumor tissue sections harvested at the end of therapy from mice receiving various treatments. Scale bar: 100 um. e) Representative photographs of lung tissues in the endpoint of antitumor therapy, the red circle indicates the lung metastatic foci. f) body weight changes of mice after different treatments. g) Serum levels of alanine transaminase (ALT), aspartate transaminase (AST), creatinine (CR), and blood urea nitrogen (BUN) in mice measured in the end of therapy. ^*^ *p* < 0.05, ^****^ *p* < 0.0001.

The therapeutic effects of O_v_‐MO@CPO‐PD in vivo were further investigated by intravenously injecting O_v_‐MO@CPO‐PD into a 4T1 tumor‐bearing nude mouse model (**Figure** **5**a). When the subcutaneous tumor volume reached ≈200 mm^3^, the mice were randomly divided into six groups (*n* = 5): control, US, MO‐PD, O_v_‐MO@CPO‐PD, MO‐PD+US and O_v_‐MO@CPO‐PD+US. MO‐PD and O_v_‐MO@CPO‐PD were administered every other day at a dose of 10 mg kg^−1^, followed by US irradiation (1 MHz, 2 W cm^−^^2^, 5 min) after each injection. Without US activation, mice in the MO‐PD or O_v_‐MO@CPO‐PD groups did not exhibit an obvious reduction in tumor growth compared to the PBS group. In contrast, both MO‐PD and O_v_‐MO@CPO‐PD showed significant tumor growth inhibition under US irradiation. Among them, the O_v_‐MO@CPO‐PD+US group exhibited the strongest tumor growth inhibition (Figure 5b,c). H&E and TUNEL apoptosis staining reveal more severe tumor cell necrosis and apoptosis in the O_v_‐MO@CPO‐PD+US group compared to the control groups (Figure 5d). Additionally, it is observed fewer and smaller pulmonary metastases in the O_v_‐MO@CPO‐PD+US group compared to other groups (Figure 5e), and H&E staining of mouse lung tissue also showed a consistent tendency (Figure S14, Supporting Information), demonstrating that O_v_‐MO@CPO‐PD can effectively inhibit tumor metastasis under US irradiation. Additionally, there was no significant weight loss in the mice of the O_v_‐MO@CPO‐PD+US group during the 12‐day treatment period (Figure 5f). After the 12‐day treatment period, histological staining of the mice's heart, liver, spleen, and kidneys, along with biochemical analysis of their serum, was performed. The corresponding results show that there were no abnormal or inflammatory lesions in tissue structures in the O_v_‐MO@CPO‐PD+US group (Figure S15, Supporting Information), and no obvious abnormalities were found in serum biochemical indices (AST, ALT, BUN, CR) (Figure 5g), indicating that the liver and kidney functions of the mice were not affected. Next, fluorescence imaging experiments were conducted to evaluate the tumor‐targeting ability of nanospheres after intravenous injection of Cy5.5‐O_v_‐MO@CPO‐PD. The fluorescence signal could be detected at the tumor site and progressively intensified. By 72 h postinjection, the tumor site showed no detectable fluorescence signal, indicating near‐complete clearance (Figure S16, Supporting Information). These results suggest that O_v_‐MO@CPO‐PD nanospheres not only effectively accumulate at the tumor site but also exhibit good retention capabilities. Collectively, these findings confirm that the designed O_v_‐MO@CPO‐PD nanospheres, targeting tumor tissues, exhibit satisfactory anti‐tumor efficacy under US irradiation and negligible toxicity as well as excellent biocompatibility in vivo.

**Figure 6:**
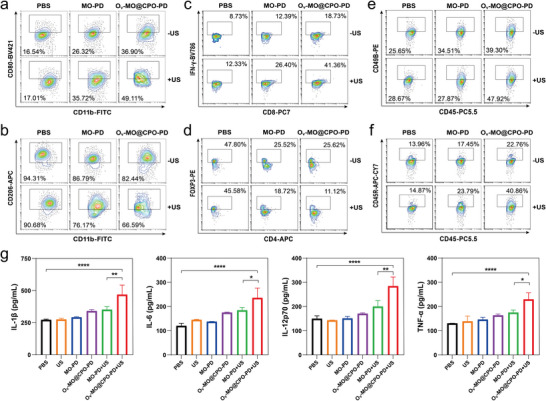
In vivo immune responses of O_v_‐MO@CPO‐PD in 4T1 tumor‐bearing mice. Representative flow cytometry images of a) M1 macrophages (CD11b^+^F4/80^+^CD80^+^), b) M2 macrophages (CD11b^+^F4/80^+^CD206^+^), c) IFN‐γ^+^CD8^+^T cells (CD8^+^IFN‐γ^+^), d) Treg cells (CD4^+^FOXP3^+^), e) NK cells (CD45^+^CD49B^+^) and f) B cells (CD45^+^CD45R^+^) in tumor tissue after various treatments. g) Cytokine levels of IL‐1β, IL‐6, IL‐12p70, and TNF‐α in serum from mice after different treatment. ^*^ *p* < 0.05, ^**^ *p* < 0.01, ^****^ *p* < 0.0001.

**Figure 7:**
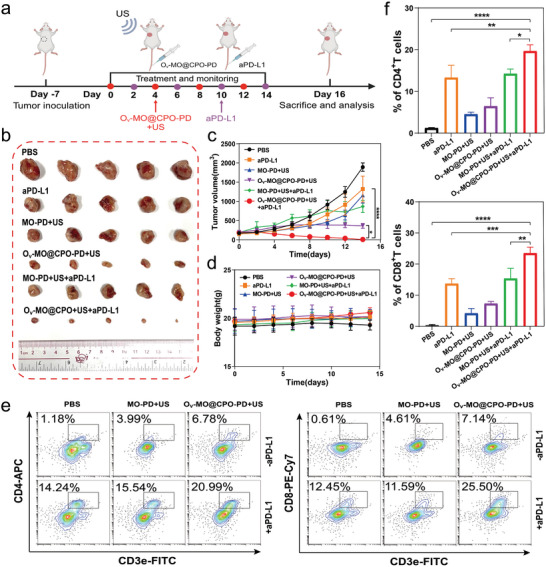
In vivo, antitumor efficacy of O_v_‐MO@CPO‐PD combined with aPD‐L1 in 4T1 tumor‐bearing mice. a) Schematic illustration of the treatment procedure for O_v_‐MO@CPO‐PD combined with aPD‐L1 antibody‐mediated synergetic cancer immunotherapy efficacy. b) Representative photographs of tumors at the end of treatments. c) Tumor growth curves and d) body weight changes of mice after different treatments. e) Representative flow cytometry images and f) quantification analysis of CD4^+^T cells (CD3e^+^CD4^+^) and CD8^+^T cells (CD3e^+^CD8^+^) in tumor tissue after different treatments. ^*^ *p* < 0.05, ^**^ *p* < 0.01, ^***^ *p* < 0.001, ^****^ *p* < 0.0001.

To further unravel the potential anti‐tumor mechanism of O_v_‐MO@CPO‐PD in vivo, we examined the immune response induced by O_v_‐MO@CPO‐PD. Tumors were isolated from mice after 12 days of treatment and prepared into single‐cell suspensions, which were analyzed by flow cytometry. The gating strategies are shown in Figure S17 (Supporting Information). Compared to the control groups, the number of immune‐activating cells (M1 macrophages, CD4^+^T cells, CD8^+^T cells, IFN‐γ^+^CD8^+^T cells, NK cells and B cells) in the O_v_‐MO@CPO‐PD+US group significantly increased, while the number of immune‐suppressing cells (M2 macrophages and Treg cells) significantly decreased in tumors (**Figures** **6**a–f and **7**e,f; Figure S18, Supporting Information). The results indicate that O_v_‐MO@CPO under US irradiation can not only repolarize M2 macrophages to the M1 phenotype but also enhance the infiltration of other immune‐activating cells into the tumor while reducing the infiltration of immune‐suppressive cells. However, we found that the number of immune cell infiltration in spleen tissue remained largely unchanged in the O_v_‐MO@CPO‐PD+US group (Figures S19 and S20, Supporting Information), which may be related to the low accumulation of O_v_‐MO@CPO‐PD in the spleen. To further assess the activation of immune response, we measured the immune‐related factors in serum. The secretion of IL‐1β, IL‐6, IL‐ 12p70, and TNF‐α in the O_v_‐MO@CPO‐PD+US group was substantially higher than in other groups (Figure 6g), indicating that the synergistic treatment effectively activates a robust immune response in vivo. In conclusion, O_v_‐MO@CPO‐PD nanospheres play a crucial role in relieving the immunosuppressive TME and effectively activating immune responses for enhanced antitumor therapeutic effects.

Finally, in order to explore the potential of combining O_v_‐MO@CPO‐PD‐mediated SDT with immune checkpoint inhibitors for enhanced anti‐tumor effect, we administered O_v_‐MO@CPO‐PD and anti‐PD‐L1 (BMS202) in a 4T1 tumor‐bearing BALB/c mouse model. When the subcutaneous tumor volume reached ≈200 mm^3^, the mice were randomly divided into six groups (*n* = 5): control, aPD‐L1, MO‐PD+US, O_v_‐MO@CPO‐PD+US, MO‐PD+US+aPD‐L1, and O_v_‐MO@CPO‐PD+US+aPD‐L1. O_v_‐MO@CPO‐PD or MO‐PD (10 mg kg^−1^) and BMS202 (20 mg kg^−1^) were separately i.v. injected every other day, with US irradiation (1 MHz, 2 W cm^−^^2^, 5 min) performed after each O_v_‐MO@CPO‐PD or O_v_‐MO@CPO‐PD injection. Tumor growth and body weight were monitored every two days during the treatment (**Figure** **7**a). Tumor growth was significantly inhibited in both the O_v_‐MO@CPO‐PD+US group and the O_v_‐MO@CPO‐PD+US+aPD‐L1 group, with the latter demonstrating a smaller tumor volume (Figure 7b,c). The body weight remained highly stable across all groups (Figure 7d), revealing the highly efficient antitumor effect and favorable biosafety of combining O_v_‐MO@CPO‐PD and aPD‐L1 therapy. Subsequently, a single‐cell suspension was prepared from the mouse tumors to analyze T‐cell subtypes using flow cytometry. The gating strategies are presented in Figure S17b (Supporting Information). We observed increased CD8^+^T cells and CD4^+^T cells infiltration numbers in tumor tissues in the aPD‐L1 and O_v_‐MO@CPO‐PD+US+aPD‐L1 groups, with the latter having a greater amount of infiltration (Figure 7e,f). These observations fully support that the combination of O_v_‐MO@CPO‐PD and aPD‐L1 under US irradiation can further facilitate the infiltration of CD8^+^T cells and CD4^+^T cells, thereby improving the effectiveness of immunotherapy and eliciting strong tumor inhibitory efficacy.

## 3 Conclusion

In summary, the O_v_‐MO@CPO core–shell nanospheres are developed as a transformable TME‐responsive sonosensitizer, which achieves highly efficient tumor therapy by sonodynamic immunotherapy. Due to the acidic degradation ability of hydroxyapatite, O_v_‐MO@CPO can dissolve the inert coating and expose active O_v_‐MO core when transferred from neutral to acidic condition, thus reinvigorating its catalytic performance to simultaneously achieve US‐stimulated ROS production, H_2_O_2_ consumption, and hypoxia alleviation. Moreover, the oxygen vacancies further boost the ROS yield of O_v_‐MO@CPO by regulating the electronic structure of the released O_v_‐MO core to not only excite more US‐irradiated electron‐hole pairs and accelerate their separation but also reduce energy barriers of catalytic reactions. As expected, O_v_‐MO@CPO effectively transforms immunosuppressive tumors to immunoreactive tumors and induces potent antitumor immunity by these multimodal synergistic effects, significantly inhibiting tumor growth and metastasis in 4T1 tumor‐bearing mice. Further combination of O_v_‐MO@CPO with an immune checkpoint inhibitor can enhance the efficacy of immunotherapy. In vivo and vitro results prove that O_v_‐MO@CPO‐driven SDT‐mediated immunotherapy has no obvious side effects. This study highlights a new strategy for designing advanced TME‐responsive sonosensitizers that have great potential in SDT‐mediated immunotherapy.

## Conflict of Interest

The authors declare no conflict of interest.

## Supporting information



Supporting Information

## Data Availability

The data that support the findings of this study are available from the corresponding author upon reasonable request.
