# Supplementary material for: Transformable Tumor Microenvironment‐Responsive Oxygen Vacancy‐Rich MnO2@Hydroxyapatite Nanospheres for Highly Efficient Cancer Sonodynamic Immunotherapy
Source: Adv Sci (Weinh). 2025 Feb 17;12(14):2414162. doi: 10.1002/advs.202414162 (PMC11984894; doi:10.1002/advs.202414162)
Supplement: Supplementary file 1 — Supporting Information [file ADVS-12-2414162-s001.docx]

Supporting Information

**Transformable Tumor Microenvironment-Responsive Oxygen Vacancy-Rich MnO_2_@Hydroxyapatite Nanospheres for Highly Efficient Cancer Sonodynamic** **Immunotherapy**

*Minxing Li,^†^ Qiyu Liu,^†^ Songzuo Xie,^†^ Desheng Weng, Jinjun He, Xinyi Yang, Yuanyuan Liu, Jinqi You, Jinghao Liao, Peng Wang,* Xihong Lu,* and Jingjing Zhao**

((Optional Dedication))

M. Li, S. Xie, X. Yang, Y. Liu, J. You, J. Liao, Prof. D. Weng, Prof. J. Zhao

Collaborative Innovation Center for Cancer Medicine; State Key Laboratory of Oncology in South China; Guangdong Provincial Clinical Research Center for Cancer; Sun Yat-Sen University Cancer Center, Guangzhou 510060, P. R. China

E-mail: zhaojingj@sysucc.org.cn

Q. Liu, J. He, Prof. X. Lu

The Key Lab of Low-carbon Chem & Energy Conservation of Guangdong Province, School of Chemistry, Sun Yat-Sen University, Guangzhou 510275, P. R. China

1. mail: luxh6@mail.sysu.edu.cn

Prof. P. Wang

Department of Emergency Medicine, Sun Yat-sen Memorial Hospital, Sun Yat-sen University, Guangzhou 510120, P. R. China.

E-mail: wangp49@mail.sysu.edu.cn

**Experimental Section**

**Chemical and materials:** All the chemicals were used directly without further purification. MnSO_4_·H_2_O (99%, M-111707-500g) was purchased from Aladdin (Shanghai, China). KMnO_4_ (≥99.5%, AD23-AR-500G) and isopropanol (IPA, ≥99.7%, GS0304) were purchased from Guangzhou Chemical Reagent Factory (Guangzhou, China). Ca(OH)_2_ (95%, C804308-500g) and polyacrylic acid (PAA, M.W ~2000, P822497-5g) were purchased from Maclin (Shanghai, China). Na_2_HPO_4_·12H_2_O (99.0%, 10020318) was purchased from Hushi of Sinopharm Chemical Reagent Co., Ltd (Shanghai, China). RPMI-1640 medium, Dulbecco’s modified Eagle’s medium (DMEM), fetal bovine serum (FBS), and 0.25% trypsin-EDTA were purchased from Gibco (Thermo Fisher Scientific, Waltham, MA, USA). CCK-8 solution was purchased from Dojindo Laboratories (Kumamoto, Japan). HIF-1α antibody was purchased from Abcam (Cambridge, MA, USA). DAPI was purchased from Solarbio Science & Technology Co., Ltd. (Beijing, China). ALT, AST, CR and BUN detection kits were purchased from BestBio (Beijing, China). DAB (SA-HRP) tunel cell apoptosis detection kit was purchased from Servicebio (Wuhan, China). Mouse inflammation array kits were purchased from RayBiotech (Peachtree Corners, GA, USA). Collagenase, hyaluronidase, and DNase were purchased from Yeasen Biotechnology (Shanghai, China). Red blood cell lysis buffer was purchased from Beyotime (Shanghai, China). Anti-CD45-PC5.5, anti-CD11b-FITC, anti-F4/80-V525, anti-CD80-BV421, anti-CD206-APC, anti-CD3e-FITC, anti-CD4-APC, anti-CD8-PC7, anti-FOXP3-PE, anti-IFN-γ-BV786, anti-CD49B-PE and anti-CD45R-APC-CY7 were purchased from BD Biosciences (San Joes, CA, USA). 2',7'-Dichlorodihydrofluorescein diacetate (DCFH-DA), Cy5.5, and BMS-202 were purchased from MedChemExpress (Shanghai, China).

**Preparation of** **MO and** **O_v_-MO@CPO:** The MO was prepared by a comproportionation reaction. 0.75 mmol of MnSO_4_·H_2_O and 3.2 mmol of KMnO_4_ were dispersed in 450 mL of deionized water under magnetic stirring, respectively. Then, the KMnO_4_ solution was quickly introduced into the MnSO_4_·H_2_O solution. After stirring for 10 min, the MO nanospheres was obtained through vacuum filtration, repeated washing with deionized water and overnight drying at 60 °C. For subsequent synthesis of O_v_-MO@CPO, 30 mg of Ca(OH)_2_ and 80 mg of PAA were dispersed in 200 mL of deionized water under magnetic stirring. Afterwards, 40 mL of MO nanospheres in water solution (1 mg mL^–1^) was dripped into the above solution. After US irradiation for 30min by using an ultrasonic cleaner (JP-030S, Skymen, Shenzhen, China), 400 mL of IPA and 2 mL of Na_2_HPO_4_ in water solution (100 mg mL^–1^) were added into the mixture. Then, the resulting solution was stirred overnight. Finally, the O_v_-MO@CPO nanospheres was obtained through vacuum filtration, repeated washing with deionized water and drying at 60 °C.

**Preparation of MO-PD and O_v_-MO@CPO-PD:** 5 mg of MO or O_v_-MO@CPO nanospheres and 25 mg of 1,2-distearoyl-sn-glycero-3-phosphoethanolamine-N-[methoxy(polyethylene glycol)-2000] (mPEG-DSPE) was dispersed in phosphate buffered saline (PBS). After US irradiation for 1 h, the mixed solution was stored in 4 °C for further use.

**Characterization:** Field-emission scanning electron microscopy (SEM, JSM-6330F), transmission electron microscopy (TEM, FEI Tecnai G^2^ F30) coupled with energy dispersive x-ray spectroscopy (EDS) mapping, X-ray diffraction (XRD, D-MAX 2200 VPC, RIGAKU), X-ray photoelectron spectroscopy (XPS, NEXSA, Thermo VG), and electron spin resonance spectrometer (ESR, A300, Bruker) were used to character the microstructure and composition of MO and O_v_-MO@CPO. The XRD was measured by Cu X-ray Kα radiation at 10^◦^ min1^–1^, and the parameters were set at 40 kV and 26 mA. The XPS was measured by Al X-ray Kα radiation, and energy calibration of the data was performed based on the hydrocarbon C 1s peak (284.8 eV) after the test. The pH was measured by the portable pH-meter (pH5 pen-based pH tester, SANXIN, Shanghai, China).

**Ca^2+^ release at different pHs:** 10 mg of MO or O_v_-MO@CPO nanospheres were dispersed in 10 mL of PBS solution at pH 6.4 and 7.4, respectively. After standing for 24 h, the solutions were filtered to obtain filtrate. Aqua regia was added to the filtrate until its volume fraction was 5%, at which point the filtrate was diluted 20 times. Then, inductive coupled plasma atomic emission spectrometry (ICP-AES, Optima8300, PerkinElmer) was used to detect Ca^2+^ concentration of each sample.

**ROS generation under ultrasound irradiation:** Objective to ·OH detection, 3 mL of MO or O_v_-MO@CPO solution (0.1 mg mL^–1^ in PBS at pH 6.4 or 7.4) was mixed with 15 μL of methylene blue (MB, 10 mM in deionized water) solution. Subsequently, the mixture was exposed to US irradiation at fixed time intervals (1 min) and then recorded by UV-vis spectra until 10 min. The process of ^1^O_2_ detection was similar except that the molecular probe was changed to 50 μL of 1,3-diphenylisobenzofuran (DPBF, 10 mM in dimethyl sulfoxide).

**Catalase-like activity:** The H_2_O_2_ (0.25, 0.50, 0.75 and 4 mM) was added into the MO or O_v_-MO@CPO in PBS solution at pH 6.4 and 7.4 (0.1 mg mL^−1^), respectively. Then, dissolved O_2_ content increment in real-time was monitored by a portable dissolved oxygen meter (JPSJ-605F, INESA). The slope of the initial linear portion is used to determine the initial reaction velocity (*V_o_*) of the O_2_ generation by both samples. According to Michaelis-Menten equation (Equation S1) and Lineweaver-Burk (double-reciprocal, Equation S2) plot, the catalytic kinetic constants of MO and O_v_-MO@CPO can be calculated.

$V_{o}=\frac{V_{max}[S]}{K_{m}+[S]}$ (S1)

$\frac{1}{V_{o}}=\frac{K_{m}}{V_{max}}\frac{1}{[S]}+\frac{1}{V_{max}}$ (S2)

Where *K_m_* is the Michaelis constant, *V_max_* is the maximal reaction velocity, *k_cat_* (Equation S3) is the catalytic constant and [*S*] is the substrate concentrations.

$k_{cat}=\frac{V_{max}}{[E]}$ (S3)

Where the sample concentration ([*E*]) is 100 μg mL^−1^, which is calculated to be about 1.15×10^−3^ M based on the molar mass of pure MnO_2_.

**Theoretical calculation method:** Our simulations were performed within the framework of density functional theory (DFT) implemented in the Quantum Espresso package (QE). The exchange-correlation energies were described using the generalized gradient approximation (GGA) with the Perdew-Burke-Ernzerhof (PBE) functional. The projected augmented wave (PAW) method was employed for the pseudo-potentials of the H, O and Mn-atoms. respectively, and the convergence criteria for the maximum force and energy oneach atom during structure relaxation were set to 0.02 eV Å^−1^ and 10^−5^ eV. The Brillouin-zone sampling was conducted using Monkhorst-Pack (MP) grids of special points with the separation of 0.04 Å^−1^. For the structural optimization and the electronic structure calculations, respectively. The lattice parameters and atomic positions were relaxed with a convergence criterion for the total energy and the ionic forces set to 10^−5^ eV and 0.02 eV Å^−1^, respectively. A semiempirical DFT-D3 force-field approach was used to include the physical van der Waals (vdW) interaction in our calculations.

**Ultrasound (US) treatment:** The ultrasound (US) treatment was performed by means of an ultrasonic physiotherapy instrument (UT1021, Nu-Tek, Hong Kong, China). To be specific, the probe of the ultrasonic physiotherapy instrument was uniformly coated with a medical ultrasonic coupling agent (Dandelion, Shijiazhuang, China), and then pressed to the position where ultrasonic treatment was required (cuvette, in vitro, in vivo).

**Cell culture:** 4T1 breast cancer cells and LO2 cells were cultured in complete RPMI-1640 medium with 10% fetal bovine serum (FBS). RAW264.7 macrophages and Hepa1-6 cells were cultured in complete DMEM medium with 10% fetal bovine serum (FBS). HUVEC endothelial cells were cultured in a special medium for HUVEC cells (Procell). The cell incubator was set at 37°C, 5% CO₂, and 95% relative humidity. The cell culture medium was refreshed every two days, and cell passage was carried out when the cell density reached about 80%.

**In vitro cytotoxicity assay:** 5×10³ HUVECs, LO2 cells, 4T1 cells or Hepa1-6 cells were seeded into each well of 96-well plates with 100 µL of the corresponding culture medium overnight. Subsequently, MO-PD and O_v_-MO@CPO-PD were added at different concentrations, and the cells were incubated for another 24 hours. Then, CCK-8 solution was added to each well, and the cells were incubated according to the manufacturer’s instructions. The absorbance of the suspension was measured by enzyme labeling instrument at 450 nm.

**In vitro ROS detection:** 1×10⁵ 4T1 cells were seeded into each well of 12-well plates with 2 mL of RPMI-1640 culture medium and incubated overnight. Subsequently, MO-PD and O_v_-MO@CPO-PD were co-cultured with the cells for 12 hours at pH 7.4 or pH 6.4, followed by incubation with 2', 7'-Dichlorodihydrofluorescein diacetate (DCFH-DA) as a ROS probe for 1 hour. Finally, the fluorescence images were captured by confocal fluorescence microscope.

**In vitro HIF-1α expression:** 2×10^5^ 4T1 cells were seeded into each confocal Petri dish with 2 mL of RPMI-1640 culture medium and incubated overnight. The cells were then treated with different formulations and incubated under hypoxic conditions for 12 hours. Hypoxic condition was induced by culturing the cells in a hypoxic atmosphere (1% O_2_, 5% CO_2_, and 94% N_2_). Afterward, 4T1 cells were incubated overnight at 4°C with the primary antibody (HIF-1α antibody). Next day, the cells were incubated with a secondary antibody for 1 hour at room temperature. Finally, the cells were stained with DAPI and imaged using confocal laser scanning microscopy (CLSM).

**In vitro flow cytometry analysis of macrophages:** 1×10⁵ RAW 264.7 cells were seeded into each well of 12-well plates with 2 mL of DMEM culture medium overnight, then stimulated with 20 ng/mL interleukin-4 (IL-4) for 24 hours to polarize them into M2-like macrophages. The M2 macrophages were then incubated with different formulations for another 12 hours at pH 7.4 or pH 6.4. Afterwards, the M2 macrophages were harvested and incubated with anti-CD11b-FITC, anti-CD80-BV421 and anti-CD206-APC antibodies. The cells were then analyzed by flow cytometry.

**In vivo tumor models and antitumor studies:** Female BALB/c nude mice aged 3-5 weeks were purchased from Guangdong Medical Laboratory Animal Center (Guangdong, China) and used according to animal protocols approved by the Institutional Animal Care and Use Committee of Sun Yat-Sen University Cancer Center (approval number: L102022023020G). 4T1 cells at a density of 1×10⁶ suspended in PBS were subcutaneously injected into the right axilla of each nude mouse. When the tumor volume reached approximately 200 mm³, the 4T1 tumor-bearing mice were randomly divided into six groups (n=5): PBS, US, MO-PD, O_v_-MO@CPO-PD, MO-PD+US and O_v_-MO@CPO-PD+US. The mice were treated with MO-PD or O_v_-MO@CPO-PD at a dose of 10 mg kg^-1^ by intravenous injection. Next, the US treatment was started immediately (1 MHz, 2 W/cm², 5 min). The tumor volume and body weight of the mice were recorded every other day. The tumor volume (V) was calculated using the formula: V = (L×W²)/2, where L and W were the longest and shortest diameters of the tumor, respectively. When the tumor volume reached 2000 mm³, all mice were euthanized and dissected.

**In vivo toxicity detection:** Blood from each mouse was centrifuged at 900 g for 15 minutes to isolate the serum. Then the concentrations of alanine aminotransferase (ALT), aspartate aminotransferase (AST), creatinine (CR) and urea nitrogen (BUN) in the serum were analyzed using the respective kits. The main organs (heart, liver, spleen, lung, and kidney) and tumor were fixed in a 4% paraformaldehyde solution. Then, hematoxylin and eosin (H&E) staining and TUNEL cell apoptosis detection were conducted after embedding in paraffin and sectioning at a thickness of 2 µm. The section images were observed using optical microscopy.

**In vivo cytokine detection:** Mouse blood was centrifuged at 900 g for 15 minutes to isolate the serum. Next, for the detection of serum cytokine including IL-1β, IL-6, IL-12p70 and TNF-α, corresponding mouse inflammation array kit were used, following the instruction provided by the manufacturer.

**In vivo flow cytometry analysis of immune cells:** The tumor tissues or spleen tissues collected from mice were digested with collagenase, hyaluronidase, and DNase, then filtered through 70 µm cell strainers to obtain single-cell suspensions. The single-cell suspension was then mixed with red blood cell lysis buffer to remove red blood cells. Cells were first stained with live/dead staining and then stained with the corresponding antibodies: anti-CD45-PC5.5, anti-CD11b-FITC, anti-F4/80-V525, anti-CD80-BV421, anti-CD206-APC, anti-CD3e-FITC, anti-CD4-APC, anti-CD8-PC7, anti-FOXP3-PE, anti-IFN-γ-BV786, anti-CD49B-PE and anti-CD45R-APC-CY7 (BD Biosciences). Finally, flow cytometry was used to analyze the immune cells. Gating was initially performed to exclude dead cells and adhesive cells. Macrophages were further gated as CD11b^+^F4/80^+^, then sub-gated as M1 macrophages (CD11b^+^F4/80^+^CD80^+^) and M2 macrophages (CD11b^+^F4/80^+^CD206^+^). T cells were further gated as CD3e^+^, and then sub-gated as CD4^+^T cells (CD3e^+^CD4^+^) and CD8^+^T cells (CD3e^+^CD8^+^); Treg cells were further gated as CD4^+^Foxp3^+^; IFN-γ^+^T cells gated as CD8^+^IFN-γ^+^. NK cells gated as CD45^+^CD49B^+^; B cells gated as CD45^+^CD45R^+^. The percentages of positive cells relative to the total cell number were calculated.

**In vivo biodistribution:** 4T1 cells (1×10⁶) suspended in PBS were subcutaneously injected into the right axilla of BALB/c mice. When the tumor volume reached approximately 500 mm³, Cy5.5-loaded O_v_-MO@CPO-PD (Cy5.5-O_v_-MO@CPO-PD) was intravenously injected via the tail vein. Fluorescent biodistribution was then monitored using an IVIS spectrum imaging system at specified time points to track the accumulation and distribution of the nanospheres.

**In vivo antitumor studies of SDT combined with anti-PD-L1:** 4T1 cells at a density of 1×10^6^ suspended in PBS were subcutaneously injected into the right axilla of each female BALB/c mouse. When the tumor volume reached approximately 200 mm^3^, the 4T1 tumor-bearing mice were randomly divided into six groups (n=5): PBS, aPD-L1, MO-PD+US, O_v_-MO@CPO-PD+US, MO-PD+US+aPD-L1 and O_v_-MO@CPO-PD+US+aPD-L1. Ultrasound activation (1 MHz, 2 W/cm², 5 min) was performed after intravenous injection of O_v_-MO@CPO-PD or MO-PD (10 mg/kg). Then, the anti-PD-L1 (BMS202 20 mg/kg) was injected into the tail vein of the mice the next day. The tumor volume and body weight of the mice were recorded every other day. Tumor volume (V) was calculated according to the formula: V = (L×W^2^)/2, where L and W were the longest and shortest diameters of the tumor, respectively. When the tumor volume reached 2000 mm^3^, all mice were euthanized and dissected.

Statistical analysis: All data were presented as the mean ±SD. Statistical analysis was calculated by using one-way ANOVA with GraphPad Prism 9.0 software. The statistical significance was defined as *P< 0.05, **P<0.01, ***P<0.001, ****P<0.0001, ns P>0.05.
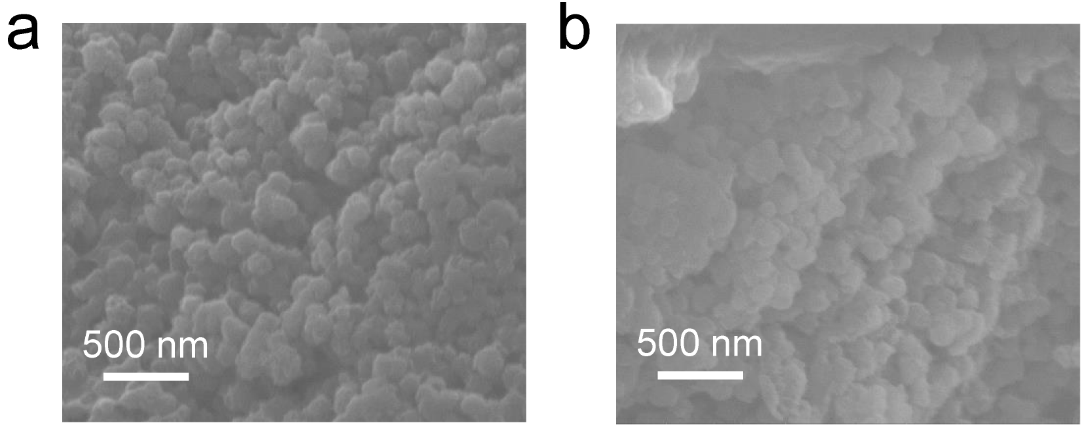


Figure S1. SEM images of (a) MO and (b) O_v_-MO@CPO.


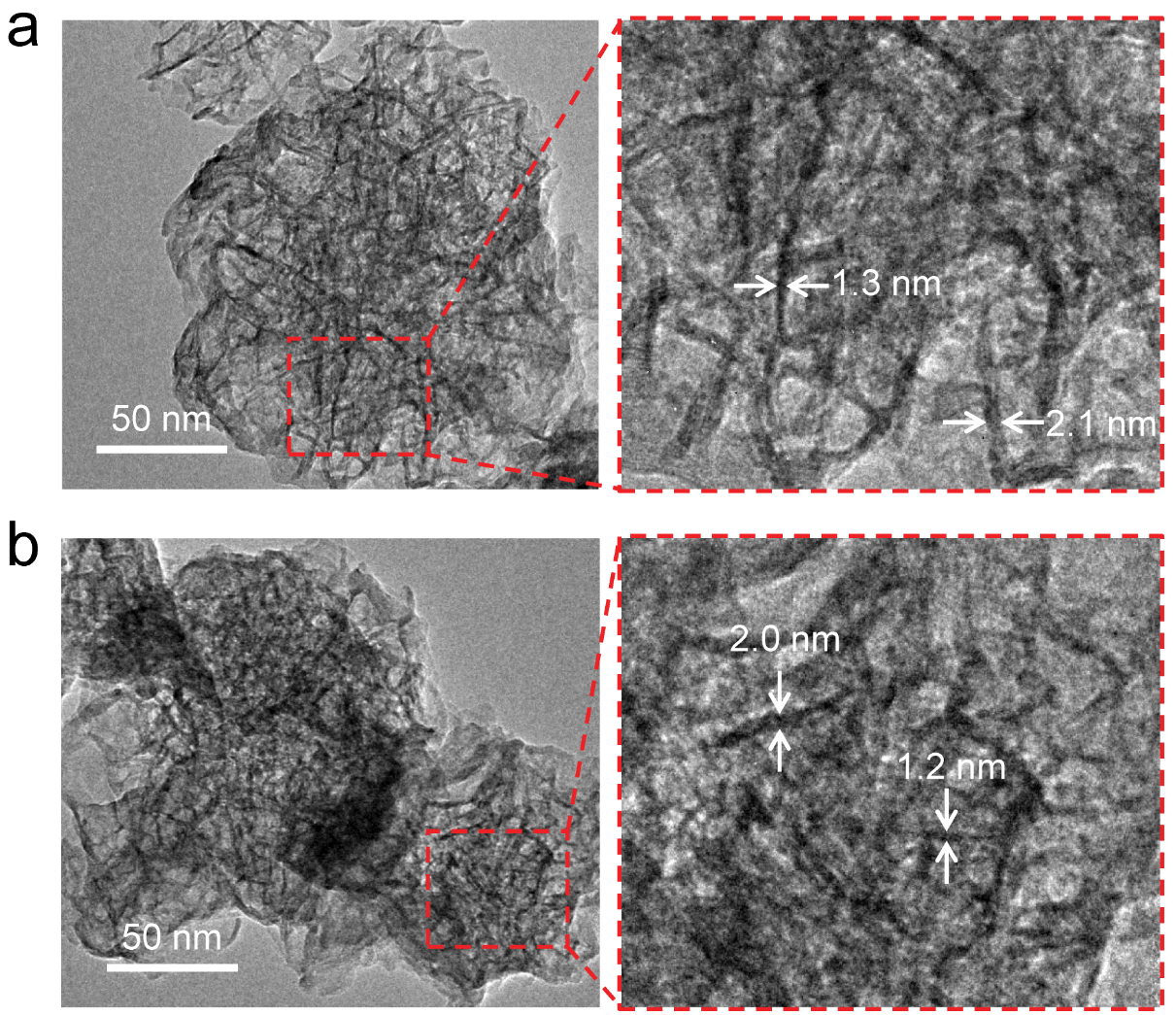


**Figure S2.** TEM image of (a) MO and (b) O_v_-MO@CPO.


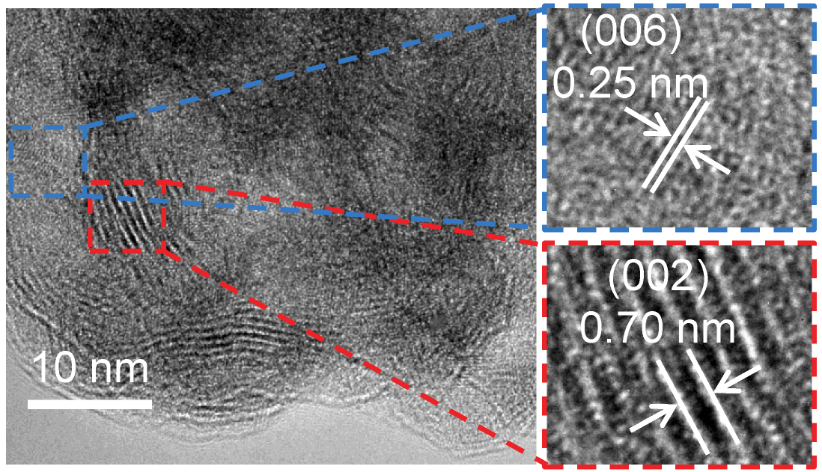


**Figure S3.** HRTEM image of MO.


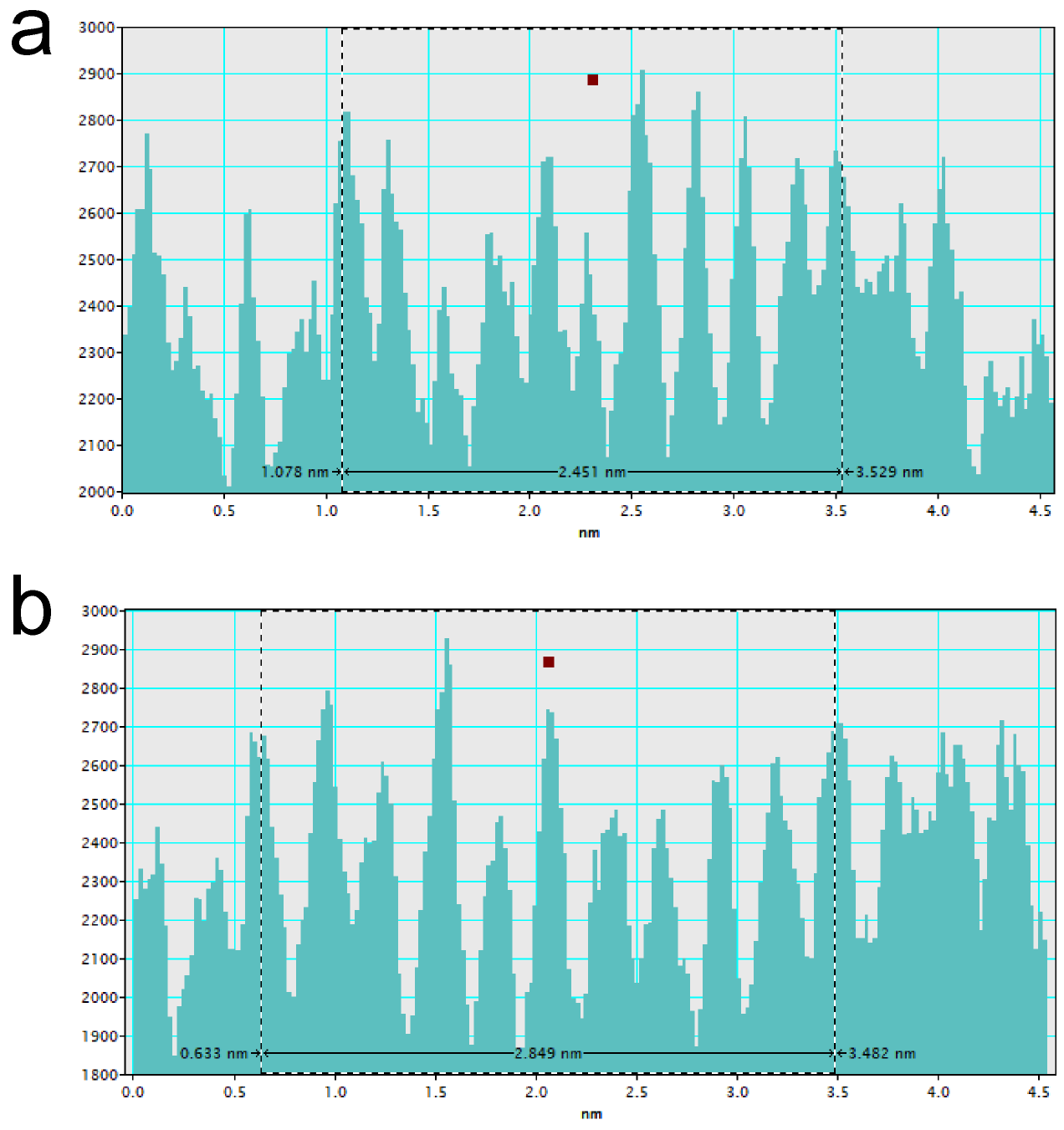


**Figure S4.** The contrast line profiles plotted according to the HRTEM images of O_v_-MO@CPO in Figure 1c.


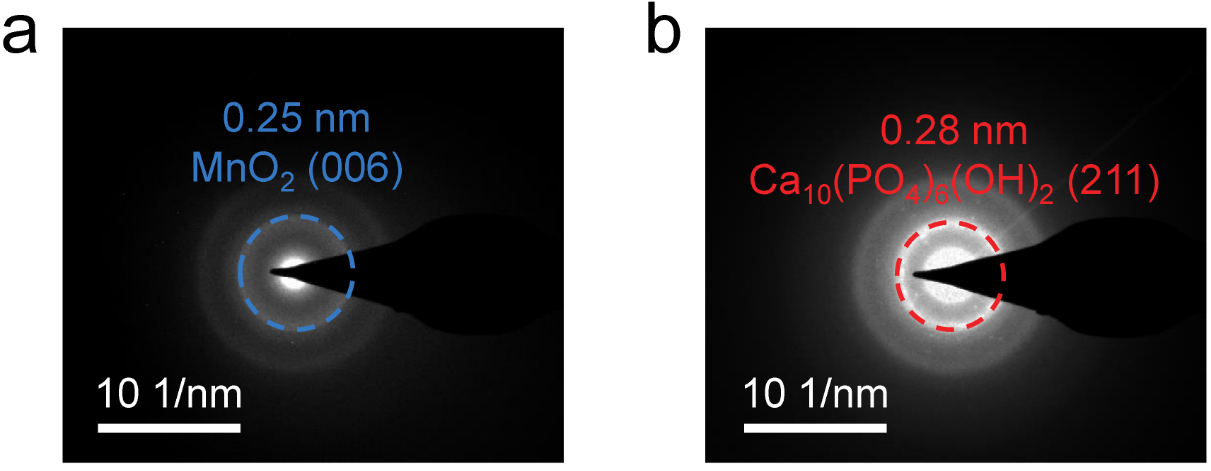


Figure S5. SAED pattern of (a) MO and (b) O_v_-MO@CPO.


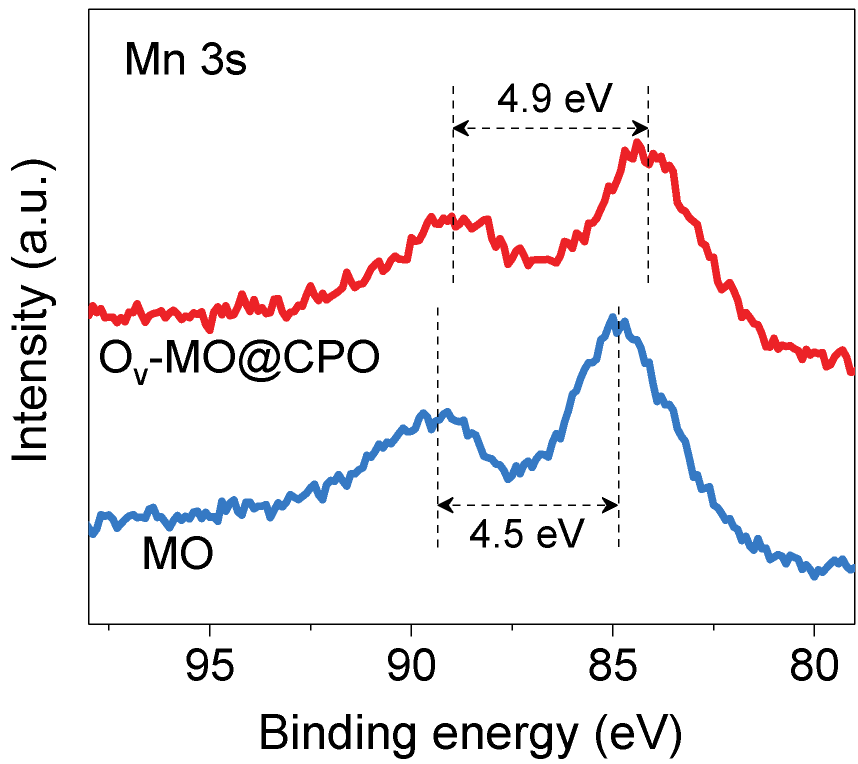


**Figure S6.** Mn 3s core-level XPS spectra of MO and O_v_-MO@CPO.


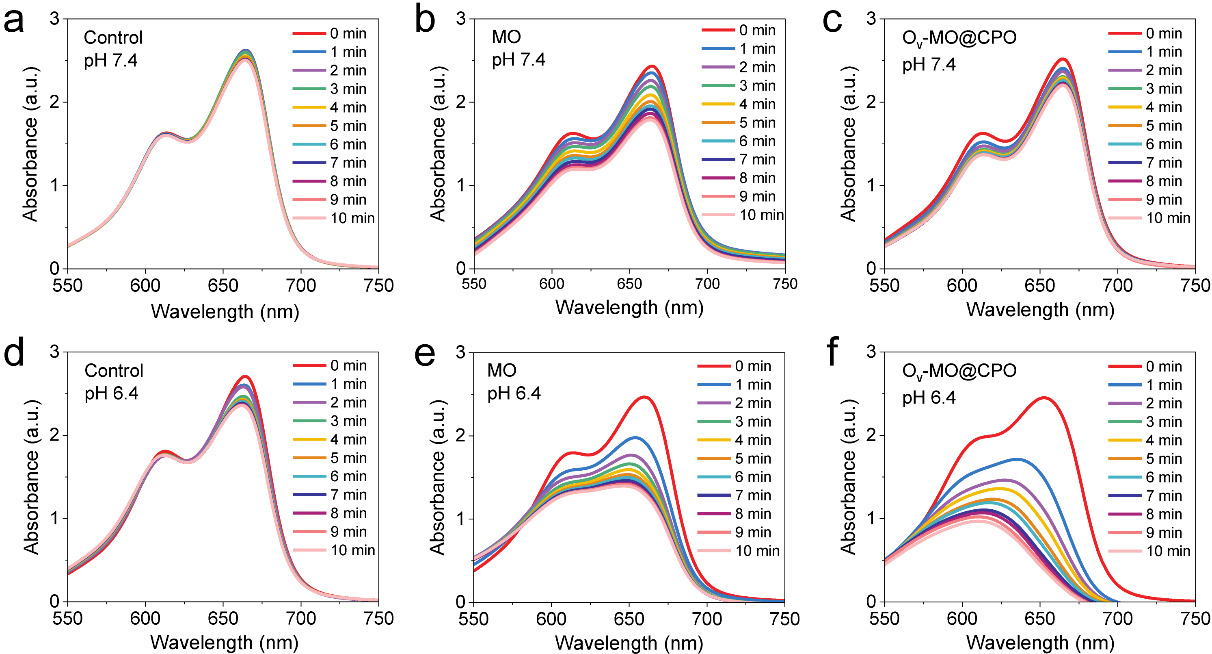


**Figure S7.** Time-dependent UV-vis absorbance spectra of MB at pH 7.4 (a) without sonosensitizer (control) and in the presence of (b) MO and (c) O_v_-MO@CPO under US irradiation. Time-dependent UV-vis absorbance spectra of MB at pH 6.4 (d) without sonosensitizer (control) and in the presence of (e) MO and (f) O_v_-MO@CPO under US irradiation.


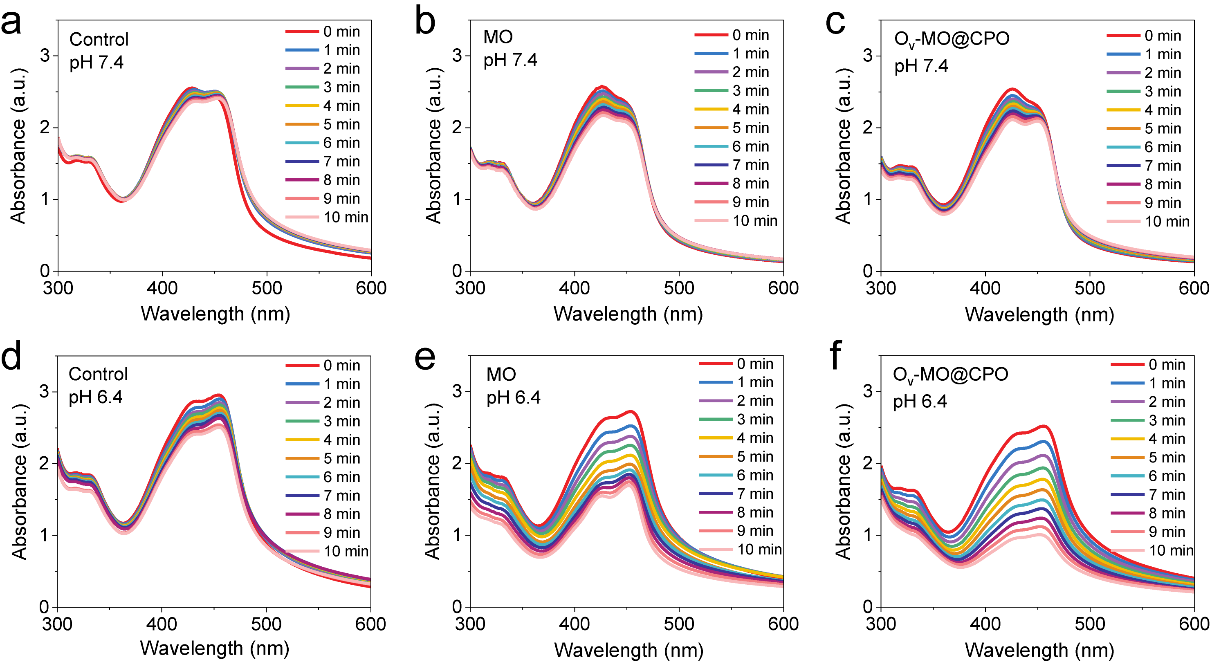


**Figure S8.** Time-dependent UV-vis absorbance spectra of DPBF at pH 7.4 (a) without sonosensitizer (control) and in the presence of (b) MO and (c) O_v_-MO@CPO under US irradiation. Time-dependent UV-vis absorbance spectra of DPBF at pH 6.4 (d) without sonosensitizer (control) and in the presence of (e) MO and (f) O_v_-MO@CPO under US irradiation.


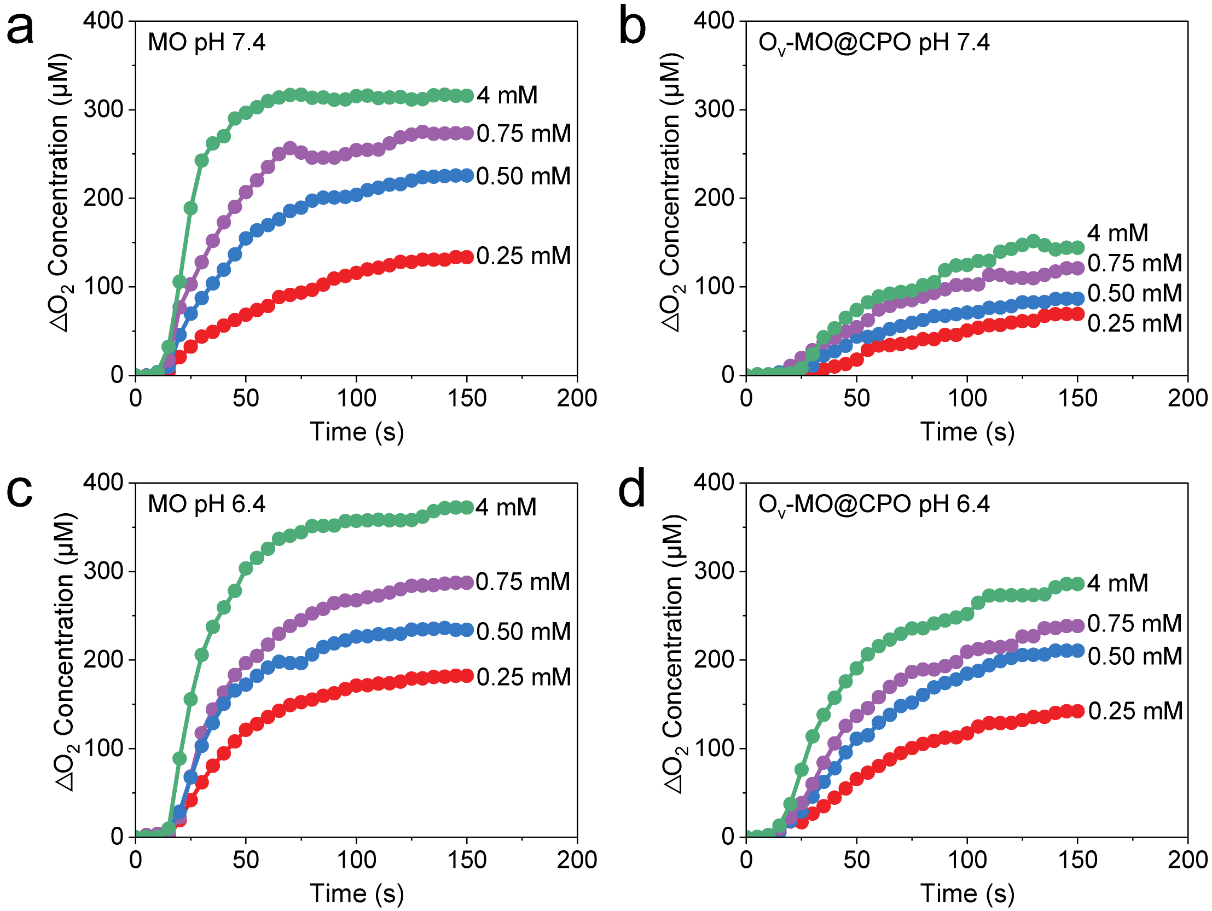


**Figure S9.** The detection of O_2_ production of (a) MO and (b) O_v_-MO@CPO with different H_2_O_2_ addition (0.25, 0.50, 0.75 and 4 mM) at pH 7.4. The detection of O_2_ production of (c) MO and (d) O_v_-MO@CPO with different H_2_O_2_ addition (0.25, 0.50, 0.75 and 4 mM) at pH 6.4.


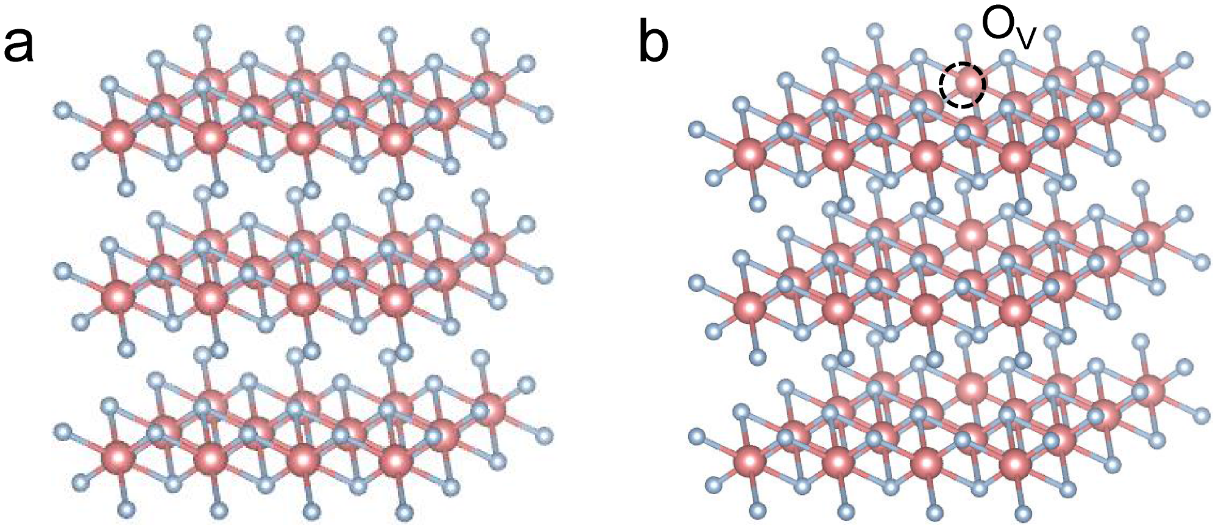


**Figure S10.** Structural model of (a) MO and (b) O_v_-MO, where blue and red balls represent O and Mn atoms, respectively.


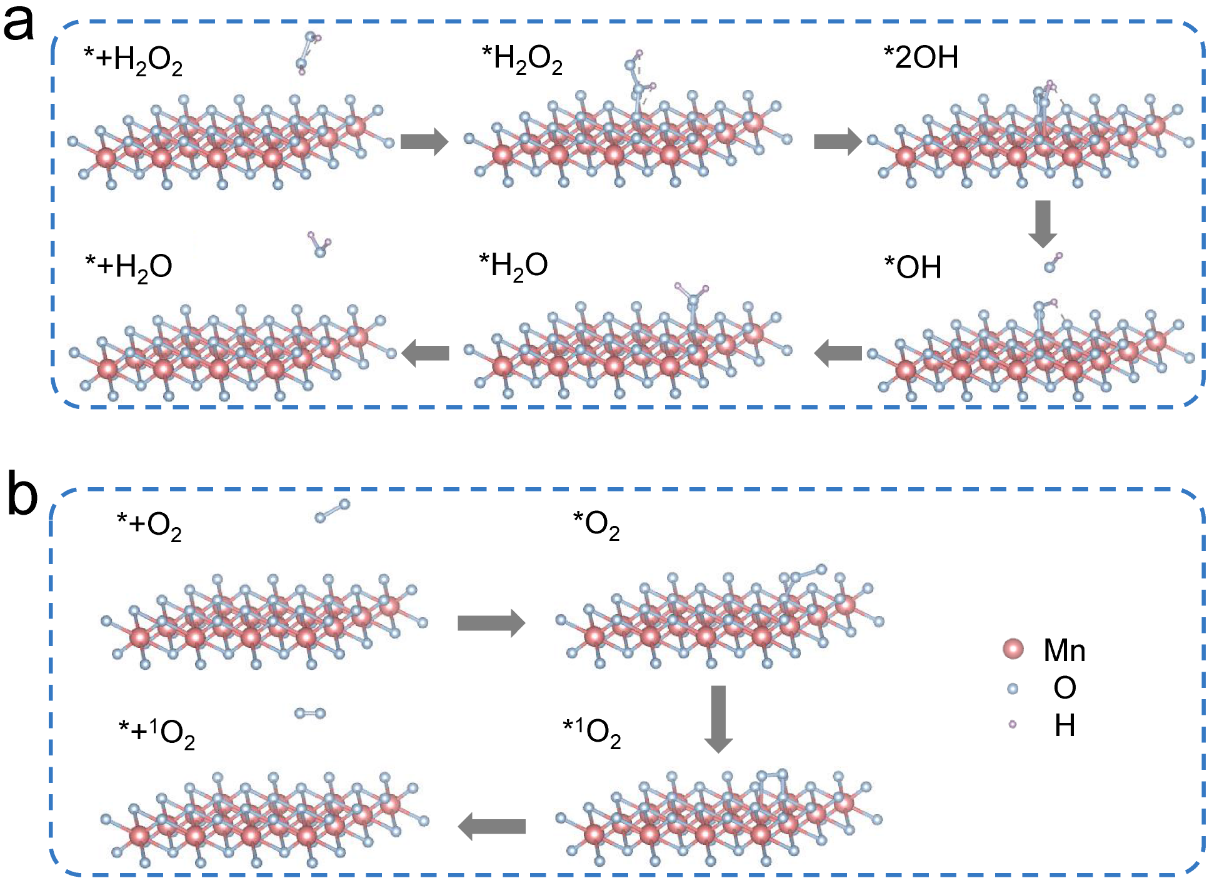


**Figure S11.** (a) ·OH and (b) ^1^O_2_ generation catalytic path of MO.


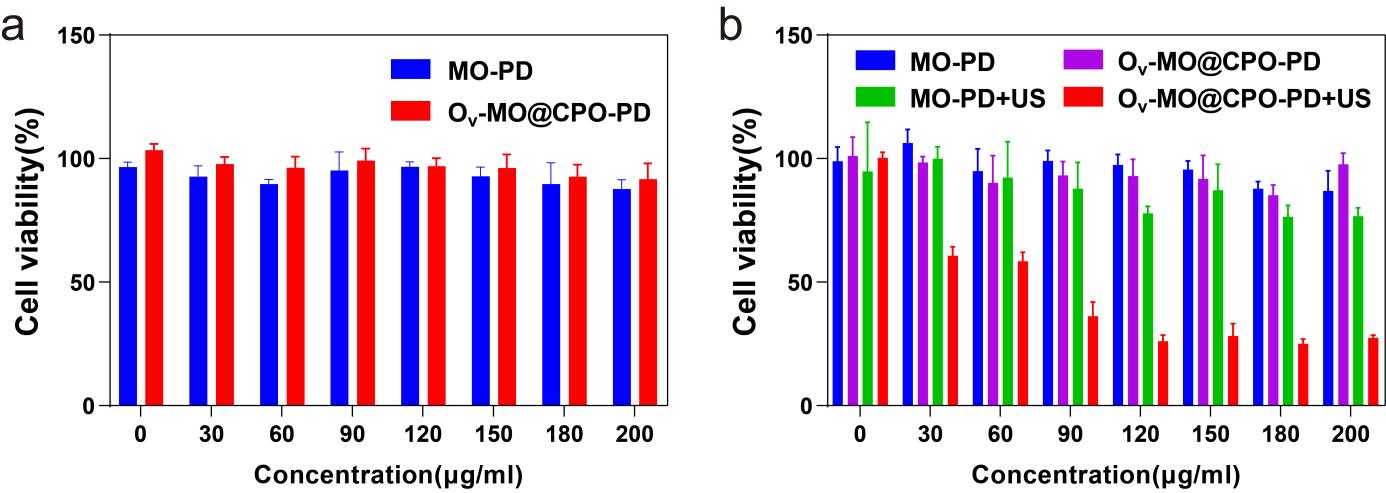


Figure S12. (a) Cell viability of LO2 cells incubated with MO-PD and O_v_-MO@CPO-PD at different concentrations. (b) Cell viability of Hepa1-6 cells incubated with MO-PD and O_v_-MO@CPO-PD at different concentrations with or without US (1 MHz, 2 W/cm², 2 min).


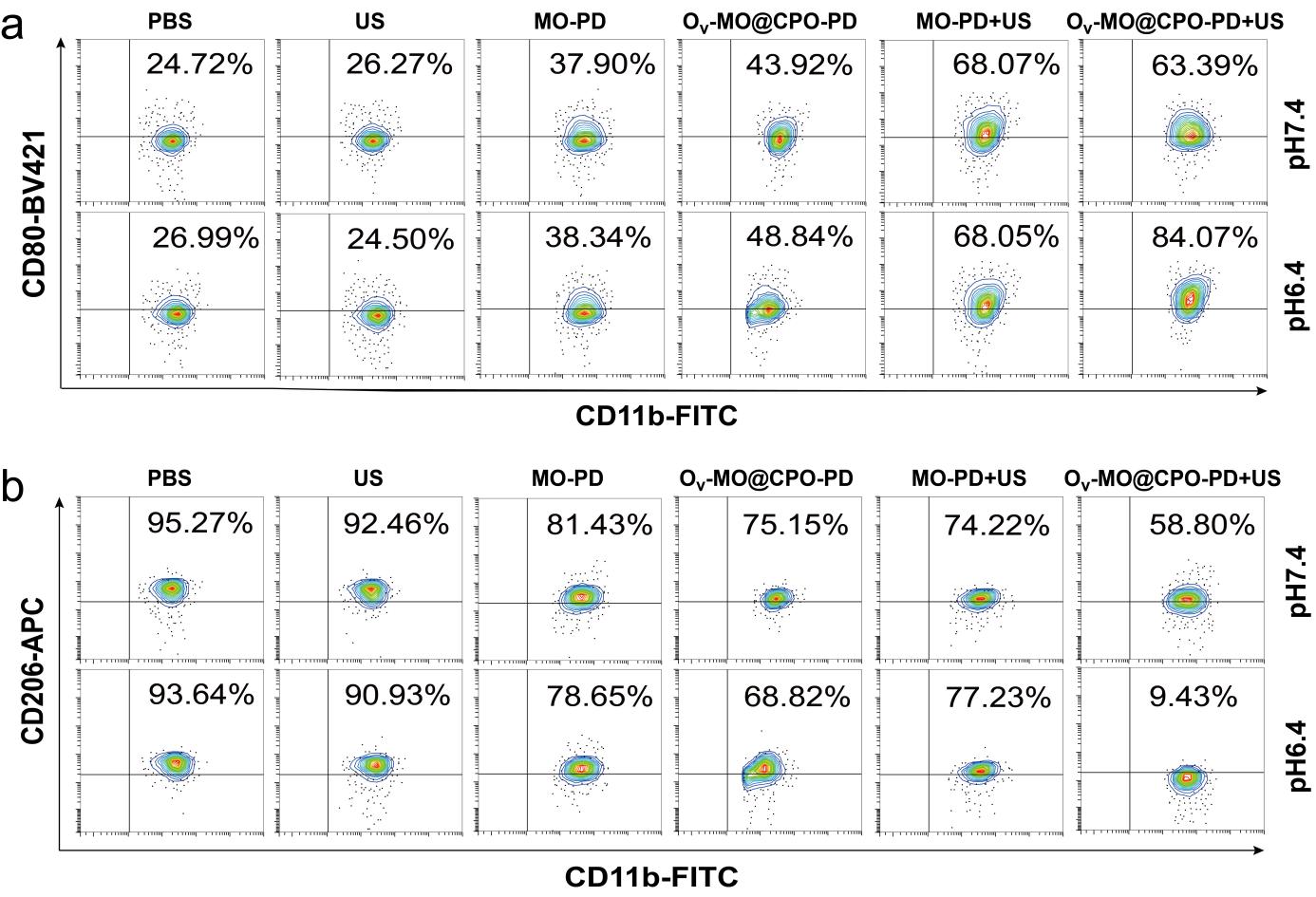


Figure S13. Flow cytometry images of (a) CD80 (M1 macrophage marker) and (b) CD206 (M2 macrophage marker) expression on Raw264.7 cell.


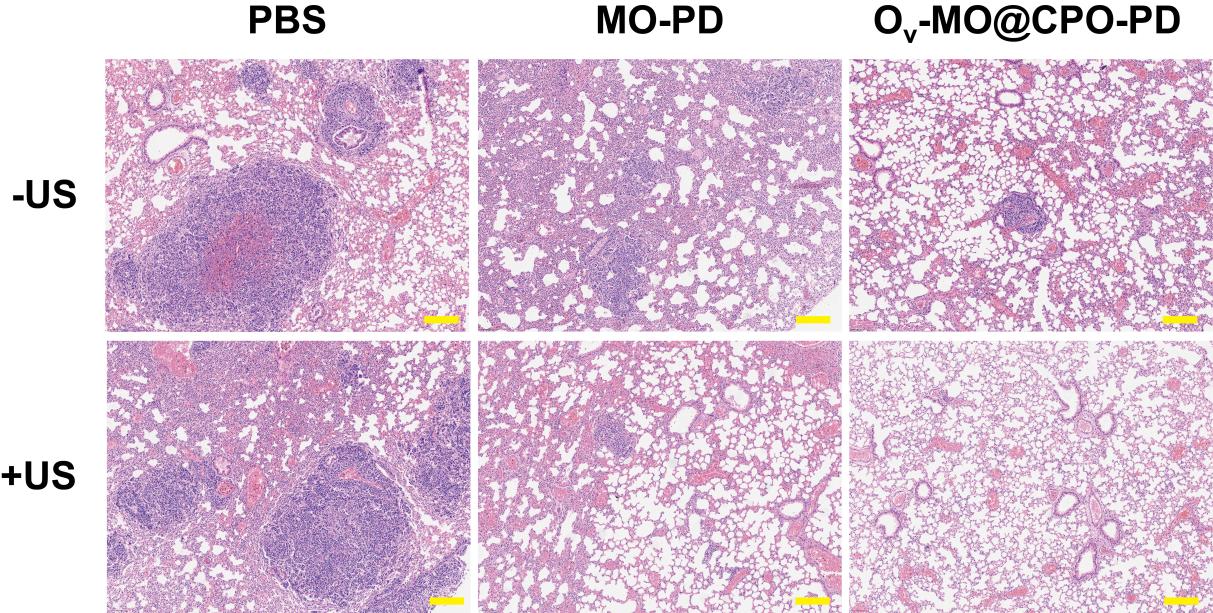


**Figure S14.** H&E analysis of lung tissues of mice after different treatments. Scale bar: 200 μm.


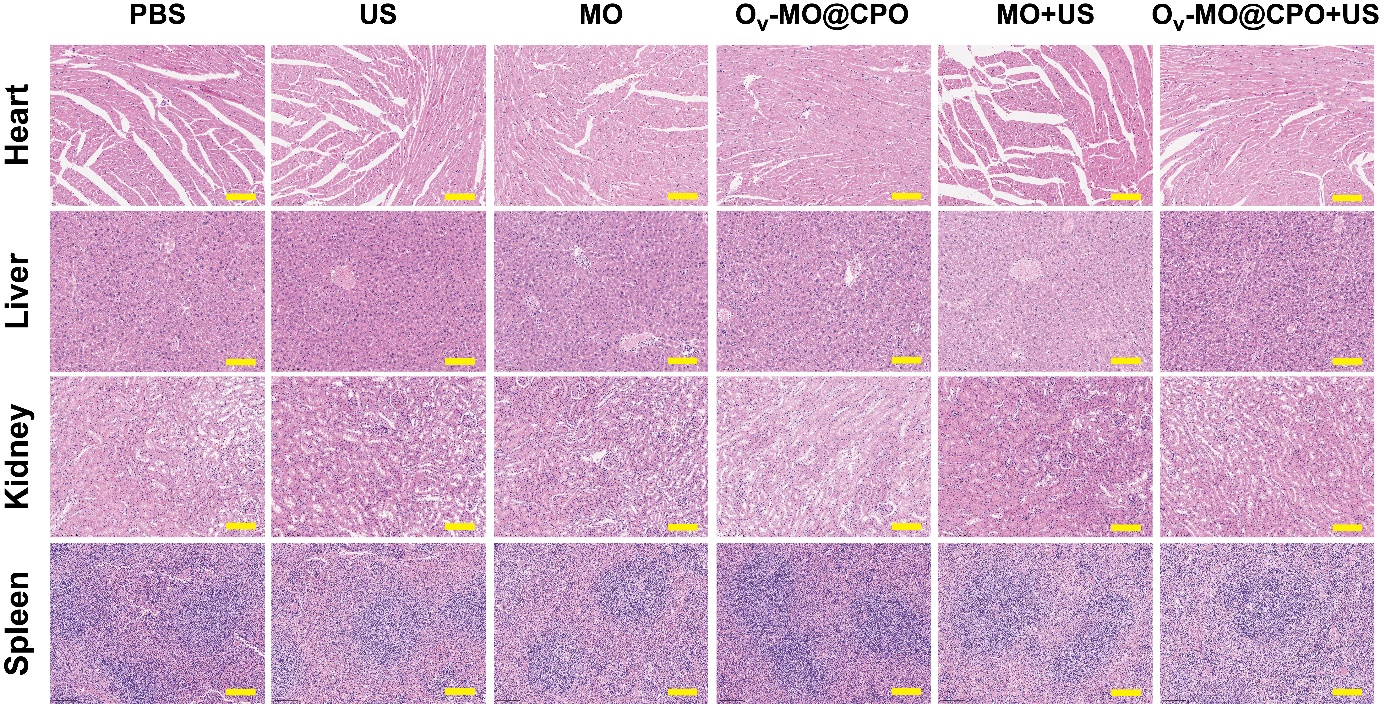


**Figure S15.** H&E analysis of major organs (heart, liver, kidney and spleen) of mice after different treatments. Scale bar: 100 μm.


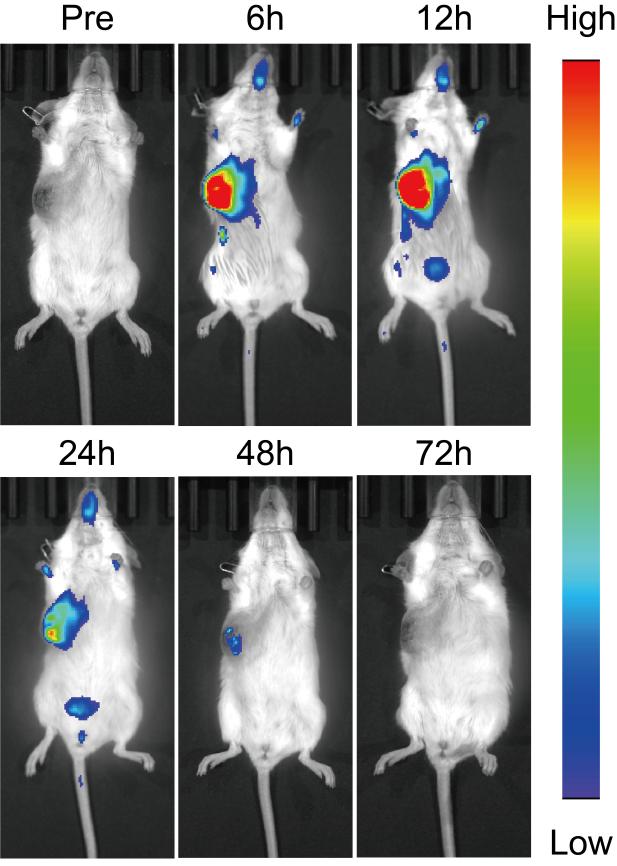


Figure S16. In vivo fluorescence imaging of 4T1 tumor-bearing mice at different times after intravenous injection with Cy5.5-O_v_-MO@CPO-PD.


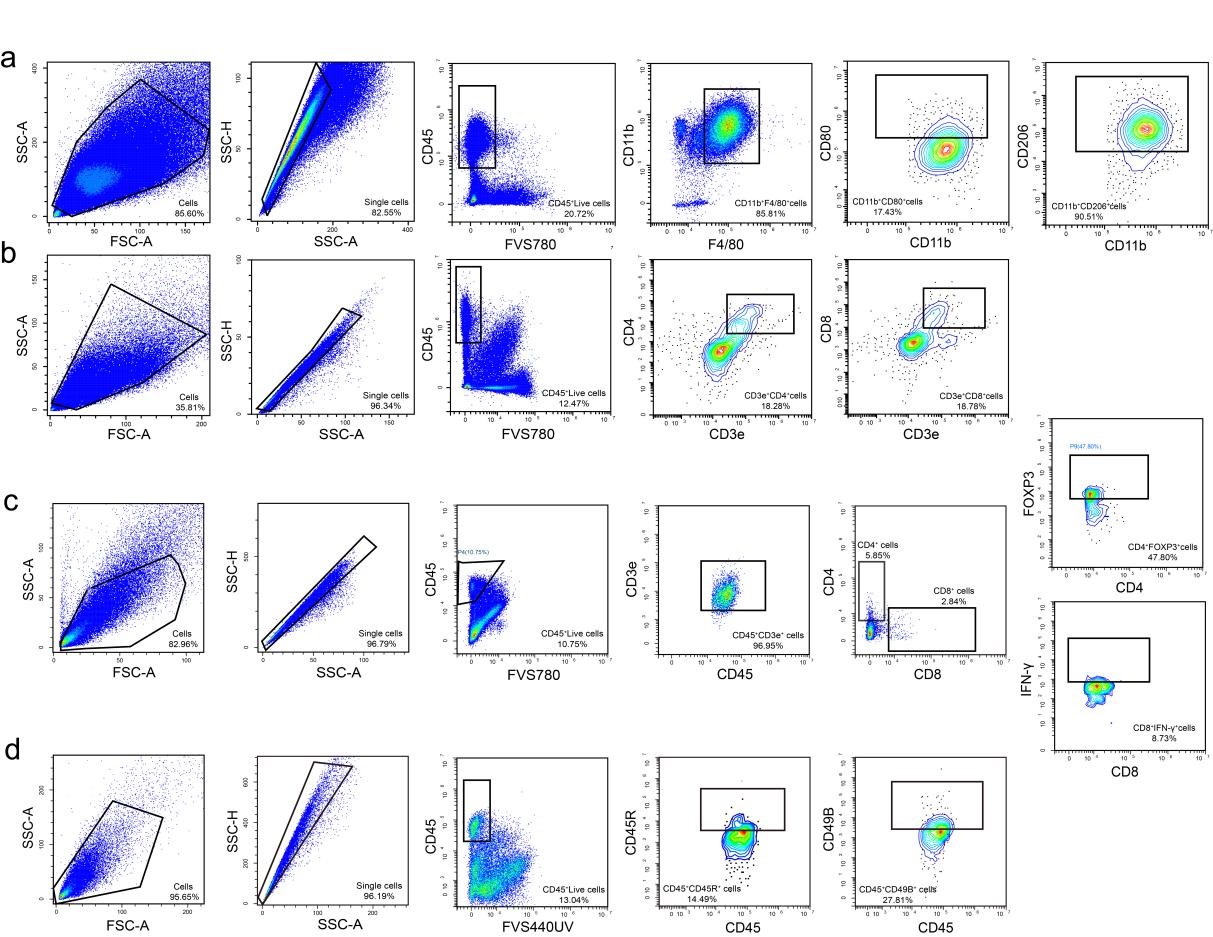


**Figure S17.** The gating strategies to sort (a) M1 and M2 macrophages, (b) CD4^+^T cells and CD8^+^T cells, (c)Treg cells and IFN-γ^+^CD8^+^T cells and (d) B cells and NK cells.


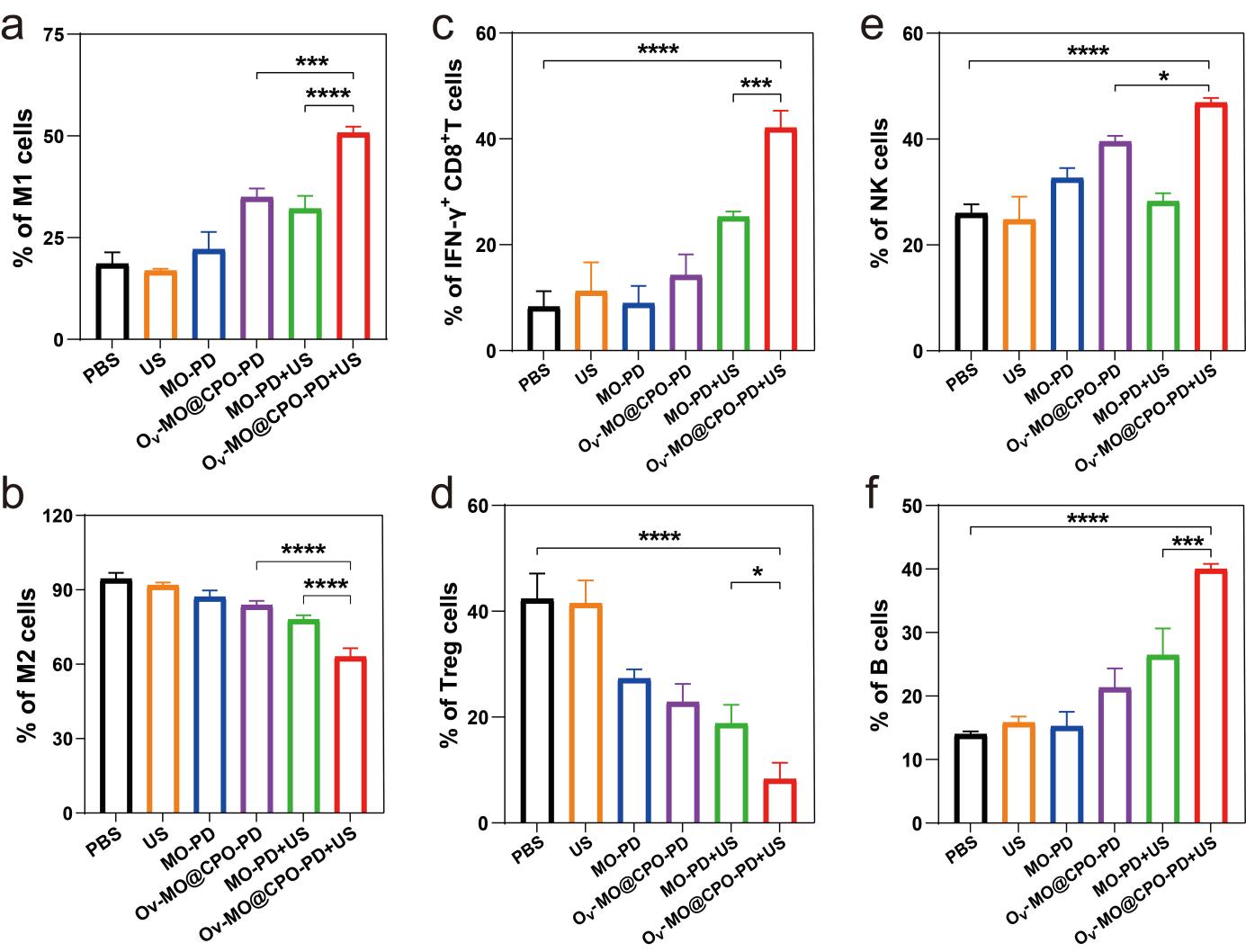


Figure S18. Representative flow cytometry quantification of (a) M1 macrophages, (b) M2 macrophages, (c) IFN-γ^+^CD8^+^T cells, (d) Treg cells, (e) NK cells and (f) B cells in tumor tissue after various treatments. *p < 0.05, ***p < 0.001, ****p < 0.0001.


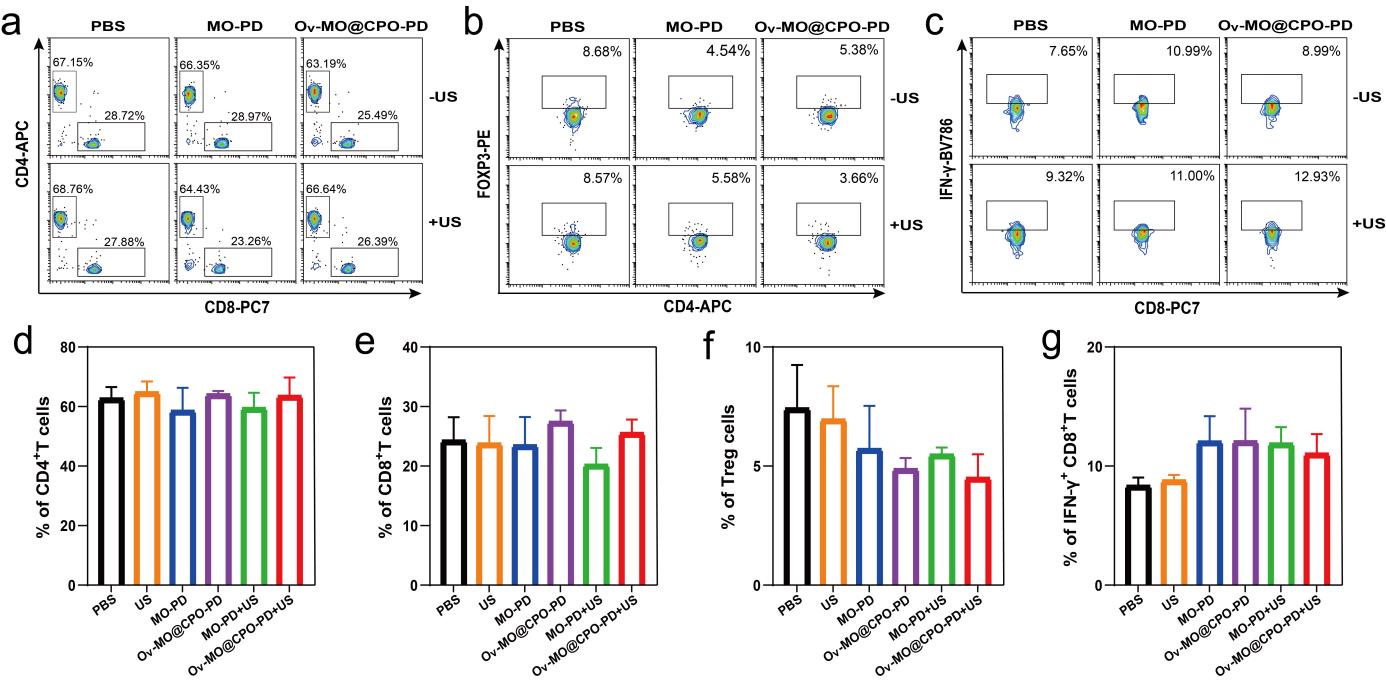


Figure S19. Representative flow cytometry images of (a) CD4^+^T cells (CD3e^+^CD4^+^) and CD8^+^T cells (CD3e^+^CD8^+^), (b) Treg cells (CD4^+^FOXP3^+^) and (c) IFN-γ^+^CD8^+^T cells (CD8^+^IFN-γ^+^) in spleen tissue after different treatments. Representative flow cytometry quantification of (d) CD4^+^T cells, (e) CD8^+^T cells, (f) Treg cells and (g) IFN-γ^+^CD8^+^T cells in spleen tissue after different treatments.


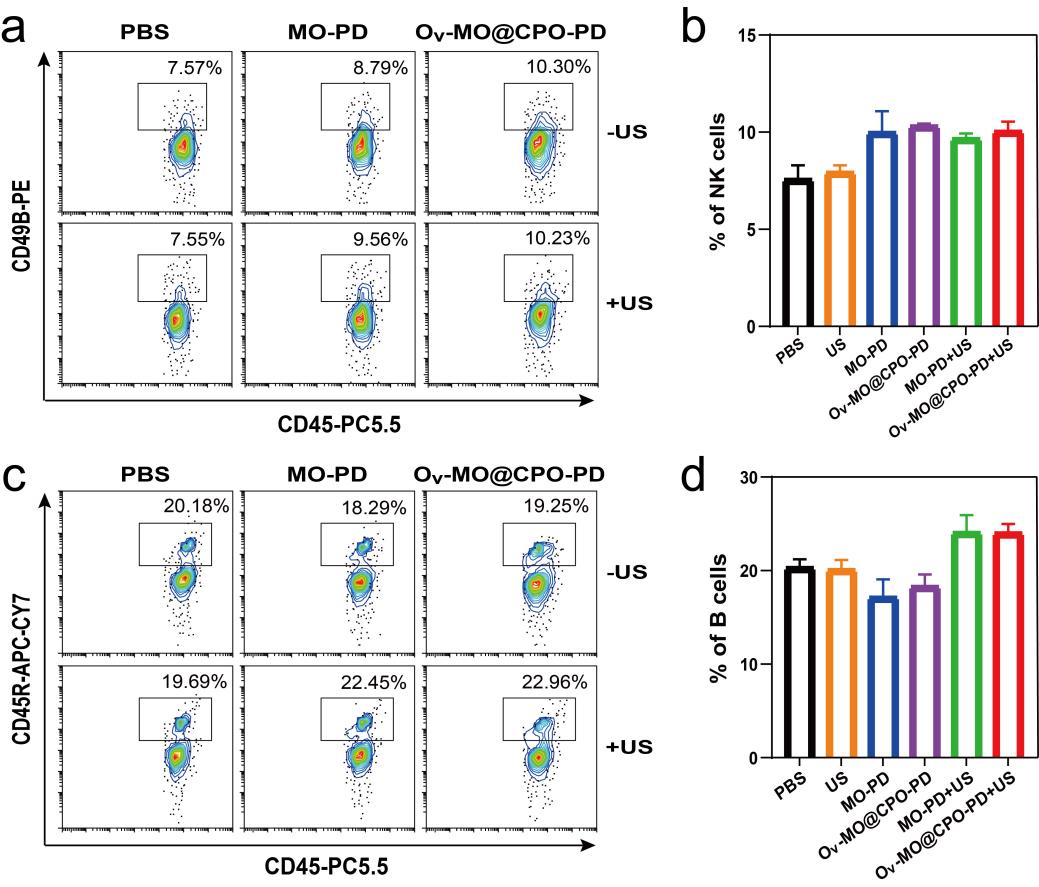


**Figure S20.** Representative flow cytometry images of (a) NK cells (CD45^+^CD49B^+^) and (c) B cells (CD45^+^CD45R^+^) in spleen tissue after different treatments. Representative flow cytometry quantification of (b) NK cells and (d) B cells in spleen tissue after different treatments.

**Table R1.** Comparison of the kinetic constants from Lineweaver-Burk plots of MO and O_v_-MO@CPO at different pH values.

| **Sample** | **pH** | ***K_m_* (mM)** | ***V_max_* (M s^−1^)** | ***k_cat_* (s^−1^)** | ***k_cat_/K_m_* (M^−1^ s^−1^)** |
| --- | --- | --- | --- | --- | --- |
| **MO** | 7.4 | 0.87 | 1.1×10^−5^ | 9.5×10^−3^ | 11.0 |
|  | 6.4 | 0.80 | 1.4×10^−5^ | 1.2×10^−2^ | 15.1 |
| **O_v_-MO@CPO** | 7.4 | 3.8 | 7.9×10^−6^ | 6.9×10^−3^ | 1.8 |
|  | 6.4 | 0.98 | 9.4×10^−6^ | 8.2×10^−3^ | 8.4 |
